# Dietary Variability Among Mountain Gorilla Groups Across Volcanoes National Park, Rwanda

**DOI:** 10.1002/ece3.71192

**Published:** 2025-05-15

**Authors:** H. Ihimbazwe, J. D. Tuyizere, L. Kayitete, D. Abavandimwe, A. K. Kamanzi Shimwa, M. L. Power, C. C. Grueter, M. Flint, J. D. Nsanzineza, A. Jonas, G. Kwibuka, D. Ishimwe, F. Ndagijimana, J. D. Hakizimana, P. Uwingeli, S. C. McFarlin, M. M. Robbins, T. S. Stoinski, W. Eckardt

**Affiliations:** ^1^ Dian Fossey Gorilla Fund Ruhengeri Rwanda; ^2^ Department of Anthropology Center for the Advanced Study of Human Paleobiology, the George Washington University Washington DC USA; ^3^ Center for Species Survival Smithsonian's National Zoo and Conservation Biology Institute Washington DC USA; ^4^ Max Planck Institute for Evolutionary Anthropology Leipzig Germany; ^5^ Department of Anatomy, Physiology and Human Biology School of Human Sciences, the University of Western Australia Perth Australia; ^6^ Rwanda Development Board Kigali Rwanda

**Keywords:** diet diversity, diet overlap, dietary evenness, food plant nutrition, habitat use, park restoration

## Abstract

Gaining a more complete understanding of a species' dietary variability is crucial to properly discern distribution, population growth trends, and conservation actions. Endangered mountain gorillas live in topographically complex forests covering a wide elevational range and diverse habitat matrices. Since 1967, mountain gorillas have been studied at high elevations in the southwest of the Volcanoes National Park (VNP) in Rwanda, where groups use different compositions of habitats and have been growing at higher rates than groups in the northeast VNP region, which is characterized by lower elevations. Building on previous efforts, we describe dietary variability among VNP mountain gorilla groups by integrating data from groups ranging in the northeast VNP. We assessed and compared nutritional components of key foods (making up 80% of the diet) to better understand whether variation in diet quality could be linked to within‐population growth differences. Feeding and ranging data were collected between November 2019 and December 2022, using long‐term monitoring data, group scans, and focal animal sampling. To compare diet quality, we combined nutritional values from newly collected food plants and previously collected and assessed food plant samples using comparable field and laboratory methods. We recorded 57 new foods for the study population. Groups in the southwest (*N* = 8) and the northeast (*N* = 4) regions of VNP used different vegetation zones, and there was high dietary variability with low diet overlap among these regions. Although northeast groups rely on more diverse diets, key foods (making up ~80% of the diet) had comparable nutrient concentrations to southwest groups. This suggests that diet quality is unlikely to be a main driver of observed heterogeneous population growth. For follow‐up research, we discuss alternative explanations linked to food distribution, biomass, and energy expenditure to access foods. Our findings add important information for future habitat suitability assessments essential for mountain gorilla conservation management and habitat restoration and expansion efforts.

## Graphical abstract

Dietary variability among mountain gorilla groups in Volcanoes National Park (VNP) was analyzed to understand how habitat differences influence diet and whether diet quality accounts for variations in population growth rates. The study found distinct diets with minimal overlap between southwest and northeast groups but comparable nutrient concentrations in key foods (~80% of the diet). This suggests that diet quality may not explain regional growth differences, prompting the exploration of alternative factors like food distribution, biomass, and energy expenditure.

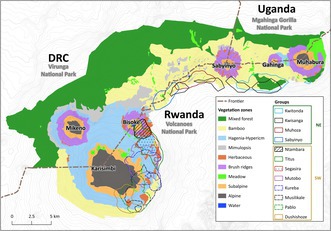

## 1 Introduction

Many primates live in complex forests with diverse habitat types, which can result in remarkable dietary variability between species (Chapman and Chapman 1990; Chapman et al. 2004). Variation in biotic and abiotic factors across different habitats strongly influences the composition of plant species and communities (Belyea and Lancaster 1999) and, thus, the availability of foods from which primates can choose. Comparative primate studies have demonstrated high dietary flexibility not only between species but also within species (e.g., Ménard et al. 2014; Tuyisingize et al. 2022a) and populations (e.g., Hanya et al. 2008; Potts et al. 2011). Intraspecific variation in food availability and dietary choices can cause considerable alterations in a species' ranging and reproduction patterns, sociality, life history, fecundity, and survival, which are important aspects shaping population dynamics (Dunbar 1990; Robbins et al. 2023; Sterck et al. 1997). Therefore, a comprehensive understanding of intraspecific dietary flexibility is critical for primate conservation management, especially for primates occupying a diversity of habitats covering a large elevational range, such as mountain gorillas.

Endangered mountain gorillas live in two small, geographically isolated Afromontane forests: the Virunga Massif of neighboring Rwanda, Uganda, and the Democratic Republic of the Congo, and the Bwindi Impenetrable National Park that connects with the Sarambwe Nature Reserve, Uganda (Hickey et al. 2020). Elevational variation between and within both forests (Virunga: ~2000–4500 m, Bwindi: ~1160–2600 m) offers gorillas a manifold mosaic of vegetation zones, which are characterized by different plant species composition and availability (McNeilage 2001; Nkurunungi et al. 2004; Rothman et al. 2007; Watts 1984). Within these vegetation zones, fine‐scaled spatial differences in environmental factors resulting from topographical complexity further contribute to the diversity of resource availability (Watts 1984). This diversity is associated with considerable dietary variation reported among gorilla groups ranging across different vegetation zones within and between populations (Ganas et al. 2004; McNeilage 2001; Rothman et al. 2007; Watts 1984; Wright et al. 2015; Vedder 1984). In addition, mountain gorillas are unevenly distributed within both forests, with gorilla and group densities varying across forest areas, and heterogeneous population growth rates occur across the Virunga Massif (Granjon et al. 2020; Gray et al. 2010, 2013; Hickey et al. 2020; Roy et al. 2014). Researchers suggested that spatial differences in gorilla density and growth rates in the Virunga Massif, evident across consecutive gorilla surveys, are linked to variations in habitat quality, topographical differences between the Virunga mountains, and the level of illegal activities and/or gorilla protection activities (Gray et al. 2010; Harcourt and Fossey 1981; McNeilage 2001; Robbins et al. 2022; Schaller 1963; Weber and Vedder 1983). However, there is a lack of comprehensive and standardized long‐term datasets on the ecology of gorillas and their habitats across all sectors of the Virunga Massif, which would provide more insights into the driving factors of heterogeneous population growth rates.

**FIGURE 1:**
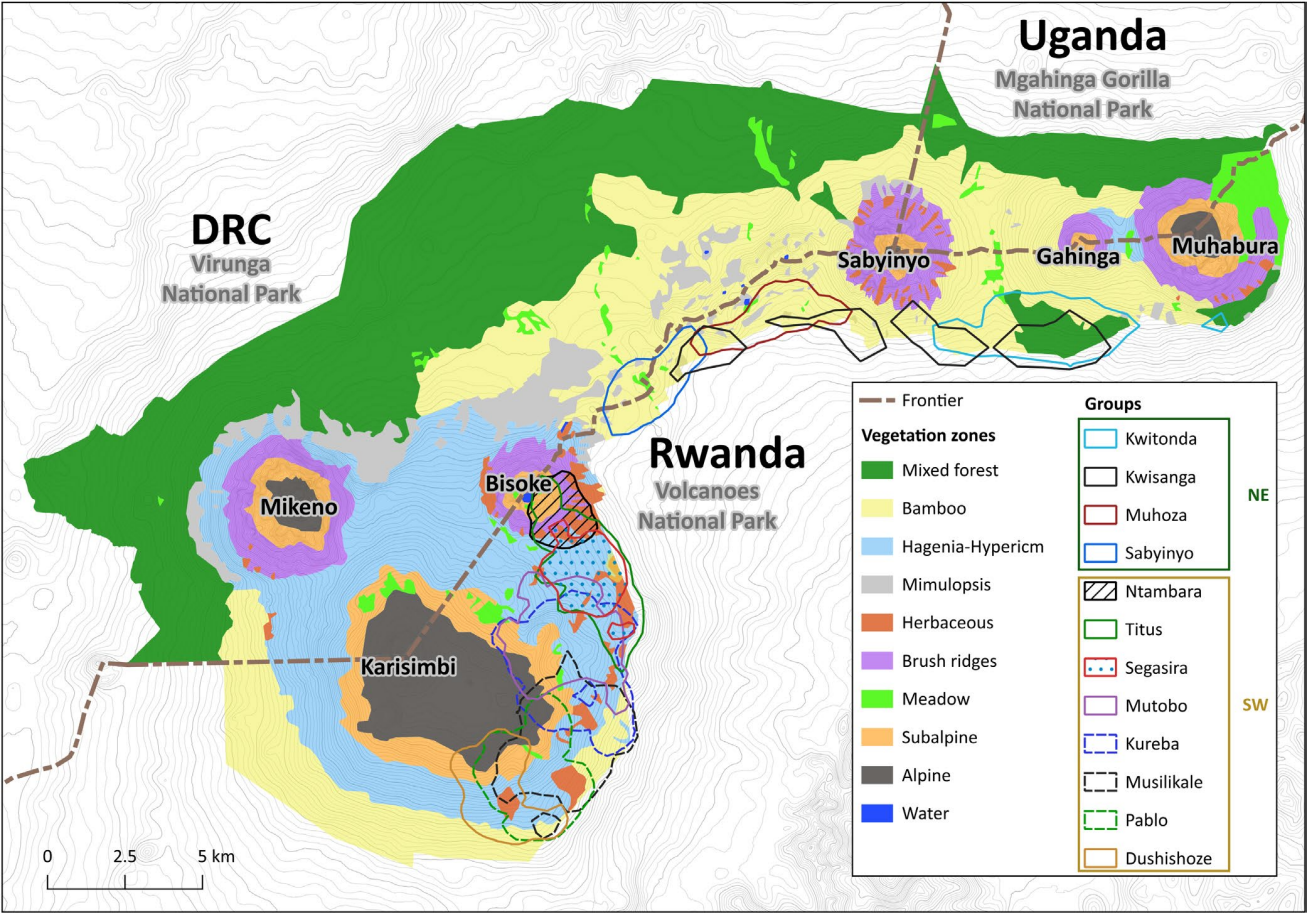
Vegetation zones in the Virunga Massif (McNeilage 1995) and home range locations of study groups in the northeast (NE) groups and the southwest (SW) group of the Volcanoes National Park in Rwanda.

Between 1989 and 2010, most of the Virunga mountain gorilla population growth was attributed to a subpopulation ranging on the slopes and in the saddle of two volcanoes, Mount Karisimbi and Mount Bisoke, in the southwest region of the Volcanoes National Park (VNP), Rwanda (see Figure 1) (Gray et al. 2010, 2013). This faster‐growing subpopulation encompasses social groups habituated for research, known as the Karisoke study population, plus the neighboring Susa group habituated for tourism (Gray et al. 2010, 2013). In contrast, the density of gorillas remained low in the eastern region and the most southern region of the VNP. However, the most recent Virunga mountain gorilla census conducted in 2015–16 showed that mountain gorilla groups increasingly use the most southern VNP region, which has been characterized as a seemingly suitable gorilla habitat (Gray et al. 2013; Hickey et al. 2019) but comparatively high rates of human disturbance (Gray et al. 2010). This gradual home range shift of groups to the south started when the number of groups of the Karisoke study population tripled between 2006 and 2009 (Caillaud et al. 2014, 2020). In contrast, the 2015–16 census reported continued low gorilla density in the most eastern VNP region, where human disturbance is comparatively low (Gray et al. 2010; Hickey et al. 2019), but vegetation is distinct and likely less suitable for gorillas, especially on the slopes of Mount Muhabura (Akayezu et al. 2019; McNeilage 1995; Weber and Vedder 1983; see Figure 1). The vegetation of Mount Muhabura stands out for its large patches of dry grasslands/meadows and was affected by two extensive fires in 1989 and 2009 (McNeilage 1995; van der Hoek et al. 2023). Furthermore, the only significant‐sized patches of Afromontane mixed forest in Rwanda occur in the eastern VNP region on the southern slopes of Mount Muhabura and Mount Gahinga. This vegetation zone covers the lowest elevations of the Virunga Massif (2000 m–2500 m; McNeilage 1995) and was transformed into agricultural land on the Rwandan side in the 1950s and 1960s (Spinage 1972) (Figure 1).

Although the Virunga mountain gorilla population has been intensively studied since 1967, almost all of today's knowledge on their behavior, feeding ecology, and life history originates from the Karisoke study population in the southwestern VNP that exhibited the highest growth rates until 2010. Numerous studies on the Karisoke study population have provided a deep understanding of their diet profile, selectivity and preferences, food intake, dietary differences between groups and habitat type, and the nutritional composition of food plants (Fossey and Harcourt 1977; Grueter et al. 2012, 2018; McNeilage 2001; Plumptre 1991; Plumptre and Harris 1995; Watts 1984; Wright et al. 2015). In a nutshell, almost 90% of their diet is made up of only six key food plant species from a total of 54 used plant species that encompass mainly perennial herbs, vines, and shrubs and fewer trees, ferns, and grasses (Grueter et al. 2012; McNeilage 2001; Watts 1984). Leaves, stems/piths, and shoots are the most important plant parts these gorillas eat, while roots, flowers, bark, and fruits are consumed less (Grueter et al. 2013; McNeilage 2001; Watts 1984). No other studied great ape population integrates fewer fruits in their diet than the Virunga mountain gorillas, which is explained by a lack of fruit trees in their high‐elevation forest (Ganas et al. 2004; Harcourt and Stewart 2007; Watts 1984). There is very little temporal variability in their diet except for bamboo shoots, which are available biannually and coincide with the rainy seasons (Fossey and Harcourt 1977; Plumptre 1995; van der Hoek et al. 2019; Watts 1984). Despite the substantial variability of plant species and communities across and within vegetation zones in the forest area of the Karisoke study population, mountain gorilla food is abundant and relatively evenly distributed across this well‐studied forest area (Plumptre 1991; McNeilage 1995; Watts 1984).

Variation in the shape, elevational gradients, vegetation zones, and microhabitats of the six volcanoes making up the Virunga Massif generates substantial ecological complexity. Yet, the only feeding study outside the Karisoke study population relied on indirect observations of a group (Group 11) ranging between Mount Bisoke and Mount Sabyinyo in a vegetation zone dominated by *Mimulopsis excellens* mixed with bamboo (McNeilage 1995, 2001). McNeilage's study also assessed gorilla food biomass in vegetation zones in the central and southwestern region of VNP, which integrated mixed forest in the DRC. However, assessing gorilla food biomass in mixed forest based on a list of foods obtained from groups ranging in other vegetation zones is problematic. Thus, more information about foods consumed across vegetation zones is needed to assess the scope of dietary flexibility in this gorilla population and to lay the foundation for future gorilla food biomass across the forest.

Our study aimed to build on previous efforts (McNeilage 1995, 2001; Watts 1984; Vedder 1984, 1989) to describe dietary variability among Virunga mountain gorilla groups using different compositions of vegetation zones, including mixed forest, integrating 12 social groups spread across the VNP between the slopes of Mt. Karisimbi and Mt. Muhabura (Figure 1). We predicted considerable differences in the diet, in terms of plant species and parts, of mountain gorilla groups that use different compositions of vegetation zones. For example, we predicted that the diet of the most eastern study group (Kwitonda) that uses Afromontane mixed forest is most distinct from other study groups that largely lack access to this forest type. Furthermore, we also expected to add new gorilla food plants to the existing list of known foods for the Virunga mountain gorillas.

To enable a comparison between the diets of groups studied over different periods (18 months versus 56 years), we used data collected from all groups within comparable time windows of up to 18 months between 2019 and 2022. Furthermore, we assessed and compared nutrients of key foods making up 80% of each group's diet to gain a better understanding of whether variation in the nutritional quality of diet could, at least in part, explain observed within‐population growth rate differences (Gray et al. 2010, 2013; Hickey et al. 2019). We predicted that the nutritional quality of key foods in the diet of mountain gorilla groups in forest areas to the east, which previously experienced slow population growth, is lower than that of key foods consumed by groups ranging in the southwest with previously fast population growth. Finally, we update and compile existing lists of food plants, items, and other consumed matter by mountain gorillas indicating overlaps and differences between the Virunga and the Sarambwe‐Bwindi population. This study will provide an important foundation for future studies to assess mountain gorilla food biomass, habitat suitability, effects of climate change on food plant distribution, and for conservation efforts including habitat restoration in the region.

## 2 Methods

### 2.1 Study Area and Animals

The study was conducted in the VNP (1°21′–1°35′ S, 29°22′–29°44′ E), which covers an elevational range from ~2400 m to 4507 m and is the Rwandan part of the Virunga Massif. The Virunga Massif is characterized by an Afromontane forest composed of a complex mosaic of different vegetation zones that vary markedly along the elevational gradient (figure 1; McNeilage 1995). We classified vegetation into distinct zones following McNeilage (1995) with some modifications. The “bamboo zone”, dominated by *Oldeania* (synonym: *Yushania*) *alpina*, covers most areas in the lowest elevations adjacent to the VNP border (2400–2950 m). This zone might be mixed with clearings colonized by various herbs, shrubs, vines, and tree species (hereinafter “mixed bamboo zone”). In higher elevations of the mixed bamboo zone, *Hagenia abyssinica* and *Hypericum revolutum* are the most prominent trees, whereas *Neobountonia macrocalyx* and *Dombeya torrida subsp. torrida* are found in lower elevations of the mixed bamboo zone. “Mixed forest” (2400–2550 m) is dominated by *Neobountonia macrocalyx* and *Dombeya torrida subsp. torrida*, which form an open canopy and are almost exclusively found on the slopes of Mount Gahinga and Mount Muhabura in the eastern VNP region. The “*Hagenia‐Hypericum* zone” (2750–3300 m) is mostly found in the southwestern VNP region and is dominated by these two trees between which dense herbaceous understory or grassy patches occur. Areas within this vegetation zone with low tree density and dense herbaceous understory are referred to as “herbaceous zone” (2800–3300 m). Similarly, the “brush ridge zone” (2950–3300 m) is within the elevational range of the “*Hagenia‐Hypericum* zone” along the ridges and ravines of volcanoes, where *Hagenia abyssinica* is usually absent and a shrub (*Senecio mariettae*) grows. In this study, we combined the “brush ridge zone” with the “*Hagenia‐Hypericum* zone” because they could not be reliably distinguished by observers, which may reflect vegetation changes within this elevational range hampering clear distinctions. At higher elevations (3300–3600 m), the “sub‐alpine zone” is found distinct by the presence of *Dendosenecio johnstonii*, *Lobelia stuhlmannii*, *Lobelia wollastonii*, and *Rubus kirungensis*. Grasses, mosses with few *Dendosenecio johnstonii* and eventually gravel and rocks take over in the “alpine zone” at the highest elevations above 3600 m. We also combined the “subalpine zone” and the “alpine zone” because gorillas usually avoided the rocky and pure grassy/meadow areas within the “alpine zone”. The “meadow zone” occurs across a wide elevation within the VNP and describes marshy or dry (eastern slope of Mount Muhabura) areas covered mostly with grasses and a few shrubs. Occasionally, groups ranged outside the park boundaries in the adjacent pasture or agricultural fields (hereinafter out‐of‐park).

The climate in Virunga Massif is characterized by four main seasons: the short dry season (December to February), the heavy rainy season (March to May), the long dry season (June to August), and the short rainy season (September to November). The annual precipitation in the VNP is ~1900 mm between 3000 and 3600 m elevation and declines at lower and higher elevations (van der Hoek et al. 2022).

The study included 12 habituated groups. Eight are part of the Karisoke study population located in the southwest of the VNP between Mount Bisoke and Mount Karisimbi (hereinafter SW groups) at high elevation (~2650–3880 m) that has been protected, monitored, and studied by the Dian Fossey Gorilla Fund's Karisoke Research Center since 1967. The remaining four groups range in the northeast of the park between Mount Bisoke and Mount Muhabura (hereinafter NE groups) at low elevations (~2400–2880 m) (Figure 1, Table 1) that are monitored daily by the Rwanda Development Board and were integrated into the Karisoke research program in November 2019. Each study group was observed for at least 8 months throughout at least one dry season and two rainy seasons.

**TABLE 1:** Size and composition of the 12 study groups during the 18‐month study period (November 2019 and December 2022) presented as monthly ranges (min/max) and by age class (infants: 0–3.5 years., juvenile: > 3.5–6 years., subadult: > 6–8, adults: > 8 years).

Group	Code	Group size	*N* adults	*N* subadults	*N* juveniles	*N* infants
Sabyinyo	SAB	15–19	7–10	1–2	2–4	2–5
Muhoza	MUH	14–21	9–11	0–1	0–3	5–7
Kwisanga	KSA	15–17	9–10	1–3	1–2	3–4
Kwitonda	KWI	17–35	9–22	1–4	2–7	1–6
Dushishoze	DUS	7–10	6	None	0–2	1–2
Pablo	PAB	16–24	10–15	1–4	1–2	3–5
Musilikale	MSK	20–24	12–13	0–2	2–4	5–6
Kureba	KRB	5–9	3–5	None	0–1	1–4
Mutobo	TOB	4–11	3–6	0–1	None	1–4
Titus	TIT	6–11	4–6	0–2	0–1	0–3
Segasira	SEG	6	3	2	1	None
Ntambara	NTA	12–15	8–10	0–1	0–2	2–4

*Note:* Gray rows indicate southwest groups and white rows indicate northeast groups.

During this study, three groups split, including Pablo (April 2021), Kwitonda (May 2021), and Titus (May 2022), leading to the formation of three additional groups, including Dushishoze, Kwisanga, and Segasira, respectively. Initially, the Kwisanga group ranged in the most eastern region of the VNP between Mount Gahinga and Mount Muhabura where the Kwitonda group is located. The Kwisanga group drastically shifted its home range westward towards Mount Sabyinyo and Mount Bisoke in June–July 2022, which exposed this group to a different composition of vegetation zones.

### 2.2 Data Collection

Data were collected for up to 18 months between November 2019 and December 2022 within two distinct periods: November 2019 to March 2020 and December 2021 to December 2022. Data collection stopped when all research activities in the VNP ceased due to the COVID‐19 pandemic.

For collecting feeding data in NE groups, we used group scan sampling (Altmann 1974) with a 10‐min observation period (scan) at 20‐min intervals over 3–4 h per day, resulting in 2828 group scans for NE groups (Table 2). During the 10 min of observation, we moved through the group to locate as many group members above 3.5 years old as possible. From every detected gorilla, we recorded its activity, which was either feeding‐related (gathering, processing, ingesting food while moving or being stationary; hereon summarized as “feeding”) or another activity, and its age class (juvenile: > 3.5–6 years., subadult: > 6–8 years., adults: > 8 years). If the gorilla engaged in a feeding‐related activity, we noted the consumed food type (plant species, other solid foods, such as animal matters, milk, or feces) and the food item (bark, cuticle, flower, fruit, (dead) leaf, pith, sap, (dry) shoot, stem, (dead) wood, rhizome, stalk, leaf sheath). If plants or animal matter were consumed as a whole, like *Galium spp* or ants, we categorized the food item as “all”. During each scan, we characterized the vegetation zone in which the group was ranging. In addition to group scan sampling in NE groups, we also recorded opportunistically any new food type‐item consumed in the four groups to compile a list of foods as comprehensive as possible within a short time.

**TABLE 2:** Number of observation days, group scans and focal scans per group and location in the Volcanoes National Park.

Group	*N* days	*N* group scans	*N* focal scans
Sabyinyo	113 (3)	722 (29)	(60)
Muhoza	105 (2)	757 (14)	(29)
Kwisanga	86 (4)	626 (39)	(107)
Kwitonda	115 (9)	723 (94)	(172)
Dushishoze	69	—	1620
Pablo	143	—	1213
Musilikale	142	—	1114
Kureba	142	—	1272
Mutobo	69	—	1813
Segasira	105 (6)	(64)	2441 (129)
Titus	139 (1)	(12)	1802 (24)
Ntambara	152	—	1425

*Note:* Gray rows indicate southwest (SW) groups, and the white rows indicate northeast (NE) groups. Numbers in parentheses indicate additional data collected to investigate the effect of sampling methods (focal versus group scan sampling).

Feeding data in SW groups were collected following the Karisoke's long‐term behavior collection protocol via 50‐min sampling of a focal animal of known identity with integrated instantaneous focal scans every 10 min, resulting in 5219 focal scans for SW groups (Table 2). The focal animal was selected from a randomized list of gorilla names. During each scan, we recorded the vegetation zone in which the group was ranging and the focal animal's activity (feeding‐related or others). If the activity was feeding, we specified the food type and item consumed using the categories as outlined above for group scan sampling in NE groups. All behavioral data were collected using Animal Observer Application v1.0 (https://fosseyfund.github.io/AOToolBox/index.html) installed on iPads.

Table S5 lists all mountain gorilla food plant species names as currently accepted by two online sources, including “Plants of the World Online” (https://powo.science.kew.org/) and “World Flora Online” (https://www.worldfloraonline.org/) and their previously accepted species names used in published mountain gorilla literature.

To investigate the effect of sampling methods (focal versus group scan sampling) on food type‐item richness and diversity in the gorilla diet, two observers collected feeding data simultaneously, one using focal sampling and the other using group scan sampling as described above in six of the 12 study groups between November and December 2022 (Table 2). Both observers regularly alternated the sampling method with each other to avoid observer biases.

Whenever possible, the locations of the groups were recorded daily at the nest site of the previous night, at arrival in the group, at noon, and when the field team departed the group using handheld Garmin devices or CyberTracker software installed on Smartphones. At each location, we also recorded the vegetation zone. Because daily group monitoring and protection efforts continued throughout the COVID‐19 pandemic with adjusted regulations, locations for most groups were continuously collected between January 2021 and December 2022 with the exception of occasional days, for example, when groups were inaccessible in a ravine or had crossed into DR Congo. Night nests were also not located every day for all groups. Similarly, if the group was located after 12 p.m., the group location at noon could not be obtained.

### 2.3 Plant Collection, Processing, and Storage for Nutritional Assessment

This study compiled data on plant nutritional content from samples of key gorilla food plant species‐items across five different sampling periods (P1‐5) between 2010 and 2022 (Table 3). For SW groups, we used existing nutritional profiles of key food plant species‐items (Grueter et al. 2012; Vakiener 2022). For NE groups, we sampled key food plant species‐items that made up at least 1% of their diet for nutritional analysis using feeding data collected over the initial 4 months of this study between November 2019 and March 2020.

**TABLE 3:** Sampling effort *(N)* presented by the number of key food plant species, plant species items, and the total number of samples used for nutritional analysis, sample collector, sampling area (SW/NE—southwest/northeast region of the Volcanoes National Park), and season (LD, long dry season; LR, long rainy season; SD, short dry season; SR, short rainy season) by sampling period.

Sampling period	Collector	Area	Elevation range	Season	*N* species^1^	*N* species‐items	*N* samples (species‐item)
1: Apr–Aug‐10^2^	CG	SW	2700–3600	LR/LD	12	15	18
2: Jul‐15^3^	MV	SW	3007–3268	LD	6	8	11
3: Jul‐17^3^	MV	SW	2860–3661	LD	3	3	18
4: Jun‐Aug‐18^4^	AI, JN	SW	2694–3600	LD	6	8	61
5: Oct‐21–Aug‐22^4^	AI, JN	NE	2356–2809	SR/SD/LR/LD	21	30	89

^1^ Ferns were not identified on species level apart from *Pleopeltis macrocarpa*.

^2^ Grueter et al. 2016.

^3^ Vakiener 2022.

^4^ Shimwa 2023.

In sampling periods 1 and 5 (P1 and P5), whenever possible, plants were sampled on the same day when at least one gorilla was observed to consume the food by sampling 500–1000 g (per species‐item) from the same plant or plants of the same food in the close surroundings. For foods that were not consumed during group follows during P1 and P5 and foods collected in P2–4, samples were obtained from forest areas where gorilla groups were known to range. In P2–4, plant samples were collected along the slope of Mount Bisoke at approximately every 100 m elevation (Vakiener 2022).

All samples were collected and stored in plastic bags, protected from sunlight, and transported to the Karisoke laboratory on the day of collection. Within 24 h of collection, samples were manipulated by mimicking the food processing behavior of gorillas specific to each food. After, the fresh samples were weighed (wet mass) and dried at 40°C–55°C in a food dehydrator or drying cabinet until the weight stopped reducing for shipping. Samples that could not be processed upon arrival at the laboratory were weighed, frozen at −20°C, which stops plant metabolism and degradation of nutrients like drying (Ortman et al. 2006), and processed the following morning. All samples were shipped for analysis to the nutrition lab of the Smithsonian's National Zoo and Conservation Biology Institute, U.S.A., except for samples from the first sampling period (P1), which were sent for analysis to the Leibniz Institute for Zoo and Wildlife Research in Berlin, Germany.

### 2.4 Data Analysis

We excluded feeding data from gorillas that consumed unknown foods. However, records of gorillas consuming foods that could not be identified but were distinguished from known and other unidentified foods remained in the dataset for analysis with the food being coded for future identification. Plants that could not be reliably identified at the species‐level were analyzed at genus‐level (*Rubus* spp., *Englerina* spp., and *Lactuca* spp.). We also combined species of genera *Carduus*, *Afrocarduus*, and *Cirsium* to “thistles” and all fern species to “ferns”, which excluded *Pleopeltis macrocarpa*. If a gorilla mixed plant items from different plant species, such as bamboo shoot with leaves of *Rubus* spp., both plant species‐items (hereafter food type‐item) were added to the total of feeding observations for calculating the importance of each plant species‐item in the diet. In further analysis, we included all feeding observations made during group scans. However, new food type‐items that were only recorded opportunistically (outside scan periods) were included in the updated list of mountain gorilla foods.

### 2.5 Habitat Use

We calculated the percentage of time each group spent in each vegetation zone using the information about vegetation zones linked to GPS coordinates collected at night nest sites and at noon (Table S1). If the field team reached the group after noon or departed from the group before noon, we accepted group locations collected at arrival in the group or departure from the group within 1 h of noon (i.e., 11 a.m. to 1 p.m.).

### 2.6 Diet Richness, Diversity, Evenness, and Overlap

**FIGURE 2:**
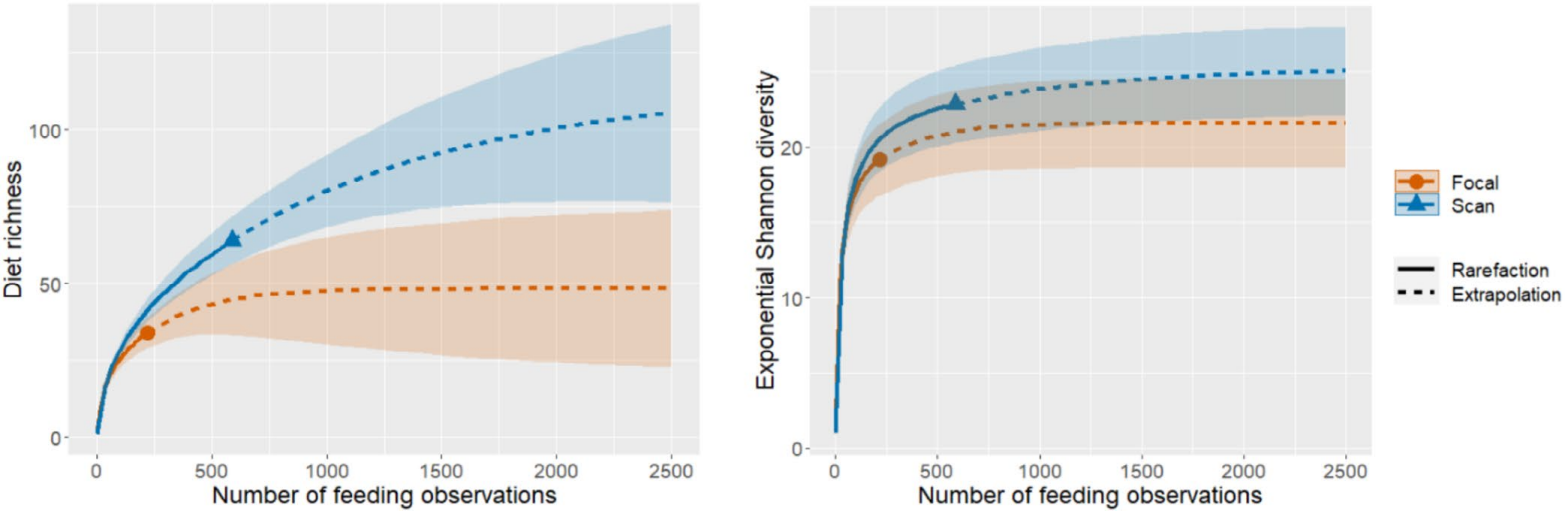
Rarefaction and extrapolation sampling curves for diet richness (*q* = 0) and exponential Shannon diversity (*q* = 1) computed from feeding data collected using focal sampling and group scan sampling.

First, we used a subset of feeding data obtained on days when focal sampling and group scan sampling were conducted simultaneously in a study group to investigate whether diet richness (the total number of different food type‐items consumed by a gorilla group) and diversity (the number of different food type‐items consumed by a gorilla group weighted by their proportion in the total diet) resulting from focal sampling and group scan sampling provide comparable outcomes. We computed diet richness and diversity using Hill numbers of order *q* = 0 (richness) and *q* = 1 (Hill‐Shannon diversity, the mean rarity of food type‐items in the diet of a group using the geometric mean) (Roswell et al. 2021) and their associated confidence intervals (CI = 95%) based on the sample size of food type‐item combinations (e.g., *Secamone africana* leaf) using the R package “iNEXT” (Chao et al. 2014). We set bootstrap replications to 100 and the endpoint of the rarefaction/extrapolated curves at 2500, which roughly represented the highest number of feeding observations in the studied groups in the full dataset. For this initial testing, we obtained 217 feeding observations from focal sampling compared to 588 feeding observations from group scan sampling. The confidence intervals of the rarefaction and extrapolation sampling curves for food type‐item richness suggest that group scan sampling overestimates diet richness compared to focal sampling when reaching about 500 to 750 observations (Figure 2). However, both sampling methods resulted in comparable estimates for diet diversity indicated by overlapping confidence intervals of the rarefaction and extrapolation sampling curves for dietary diversity independent of sample size (Figure 2).

After, we calculated diet richness and the Hill‐Shannon diversity index using the full dataset to compare the dietary composition between groups and among groups by vegetation zone. We also calculated and compared the diet overlap among groups (the extent to which two gorilla groups share food type‐items) using the Pianka index (Pianka 1974). This index ranges from zero to one, with “0” indicating that two groups do not share any food type‐item and “1” indicating two groups consume the same food type‐items at the same proportions. Finally, we calculated the food type‐item evenness (how evenly gorillas distribute their feeding time among food type‐items) in each group's diet using the Pielou index (Pielou 1977), which is obtained by dividing the Shannon‐Wiener diversity H' index (Shannon and Weaver 1963) by the natural logarithm of the total number of consumed food type‐items. The Pielou index also ranges from zero to one, with “0” indicating maximum evenness (only food type‐item is consumed) and “1” indicating (all food type‐items are equally consumed).

### 2.7 Nutritional Analysis

Using comparable standardized protocols across both laboratories (P1: Ortmann and Bradley 2006; P2‐5: Shimwa 2023; Vakiener 2022), we measured the proportion of macronutrient in the dry matter of food plant species‐item samples, including lipids (L), indigestible carbohydrates targeting neutral detergent fiber with residual ash (NDF) and acid detergent fiber with residual ash (ADF) that are components of NDF, crude protein (CP), and inorganic matter (total ash: ASH). From these proportions of plant dry matter, we calculated (1) the total nonstructural carbohydrates (Conklin‐Brittain et al. 2006): %TNC = 1−%L−%CP−%NDF−%ASH, and (2) the metabolizable energy concentration (MEC) in kJ/g of dry matter using conversion factors previously applied to nutritional studies in mountain gorillas (Grueter et al. 2016, Wright et al. 2014): MEC kcal/g dry mass = (4kcal/g × %TNC) + (4kcal/g × %CP)+(9kcal/g × %L)+(1.6kcal/g × %NDF) where % nutrient is expressed as decimal (e.g., 41% NDF = 0.41 in the formula). Before chemical analysis, subsamples from all samples underwent a second drying process. Subsamples obtained during sampling period 1 (Table 3) were dried at 105°C overnight. Subsamples from sampling period 2–5 were dried at 100°C for 3 h, except for NDF and ADF analysis. We therefore corrected proportional NDF and ADF values by 0.961 (sampling periods 2 and 3) and by 0.93 (sampling periods 4 and 5) based on the mean dry matter obtained from the drying procedure for ash analysis. Furthermore, NDF and ADF residues from sampling period 1 are reported without residual ash, whereas NDF and ADF values from sampling periods 2–5 may contain small amounts of ash.

To compare the quality of diet among study groups, we first calculated the mean proportion of each macronutrient and the mean MEC of all food plant species‐items covering 80% of feeding observations (hereinafter key foods) of each group's diet (Table S2). For foods that were not analyzed on a plant species‐item level (e.g., *thistles* were analyzed on genus‐item level), we extracted mean values of consumed items within the higher plant‐item level. We ran a Kruskal–Wallis test to compare mean MEC (kcal/g) of key foods between study groups. For macronutrients and TNC presented as mean proportional data, we ran a general linear model with a quasibinomial error distribution and group identity as apredictor.

In a second approach, we compared the diet quality of SW and NE groups by accounting for the dietary importance of key foods in each group, acknowledging that feeding time spent on each key food does not represent food intake rates. This was achieved by multiplying the percentage of observations a group fed on each key food with its mean proportion of macronutrient and mean MEC before summing weighted mean values of all key foods for each macronutrient and MEC. If the sum of the percentage of key foods exceeded 80% in the diet (e.g., 80.5%), we reduced the percentage of the key food with the lowest importance (e.g., 3%) by the percentile difference (e.g., 3%–0.5% = 2.5%). Using independent sample *t*‐tests, we compared weighted macronutrients and MECs of SW groups with NE groups, except for TNC for which we used a Mann–Whitney *U* test.

## 3 Results

### 3.1 Habitat Use

**FIGURE 3:**
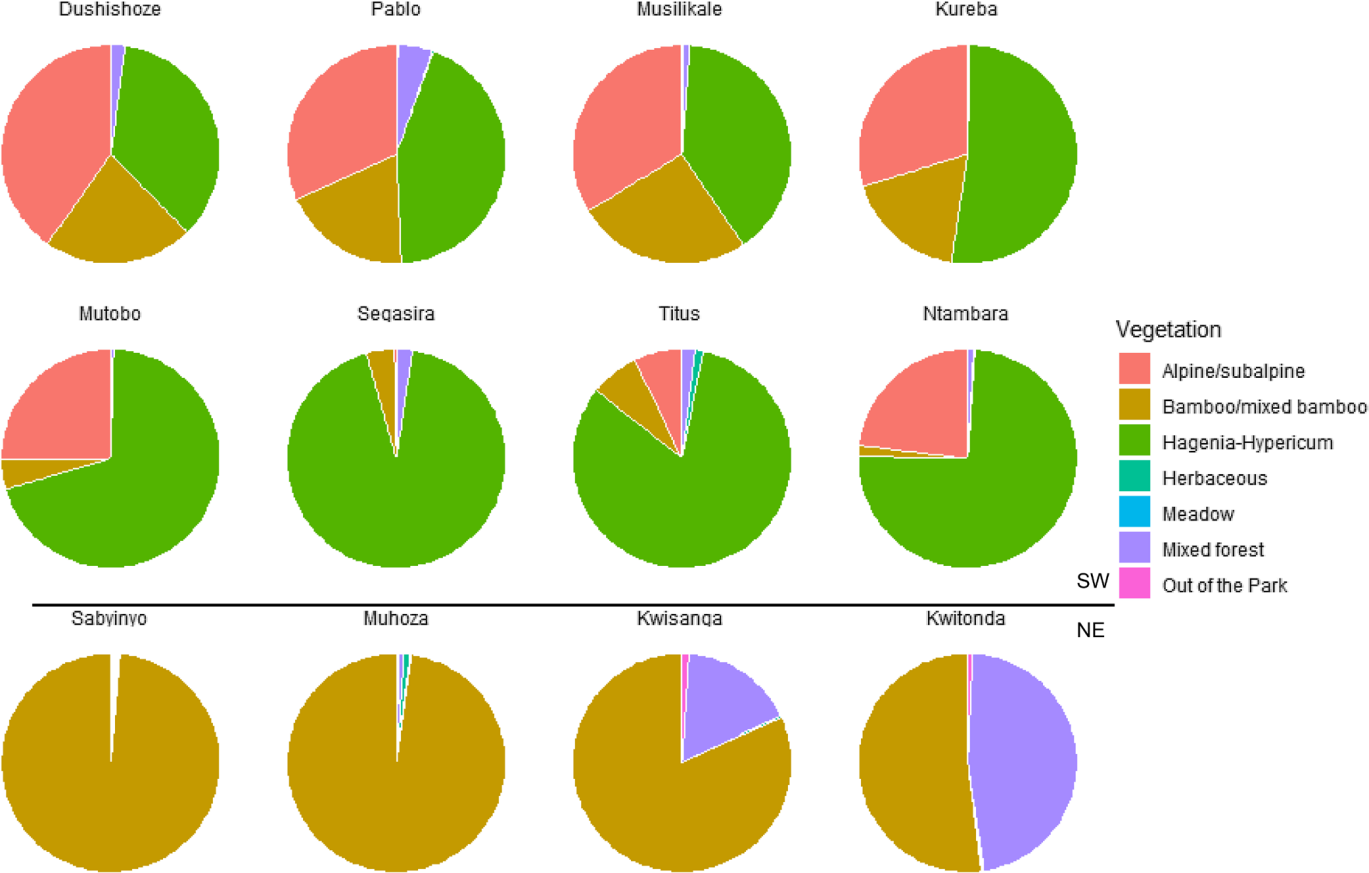
Pie charts indicate the percentage of time each study group (top and middle row: Southwest (SW) groups; bottom row: Northeast (NE) groups) spent in each vegetation zone during the study period.

SW groups predominantly used the “*Hagenia‐Hypericum* zone” and “alpine/subalpine zone”, whereas NE groups mostly occupied the “bamboo/mixed bamboo zone” (Figure 3). Only Kwitonda and Kwisanga spent substantial time in the “mixed forest zone” in addition to the “bamboo/mixed bamboo zone.” However, Kwisanga group members stopped using the “mixed forest zone” when they shifted their home range from the far east of the VNP towards Mount Sabyinyo and Mount Bisoke to the neighborhood of Muhoza and Sabyinyo groups.

**FIGURE 4:**
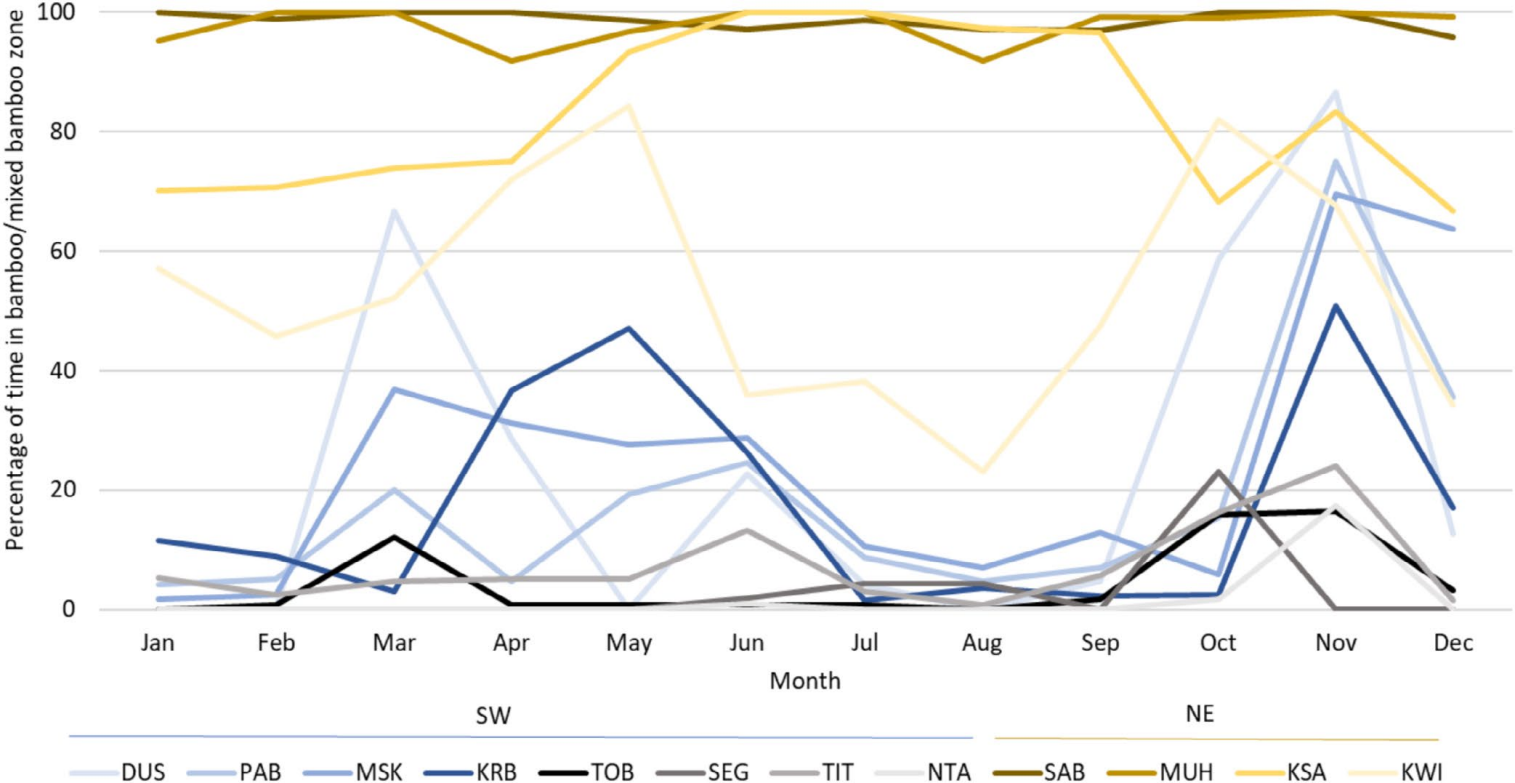
Percentage of time study groups in the southwest (SW) and northeast (NE) of the VNP spent in the “bamboo/mixed bamboo zone” each month of the year (2020–2022). SEG was not observed from January to April.

Temporal patterns of using the “bamboo/mixed bamboo zone,” where shoots occur biannually during the rainy seasons, also varied among groups from almost exclusive use (> 90% of the time across the year) to essentially never using (Figure 4). Most groups used the “bamboo/mixed bamboo zone” seasonally with peaks of varying magnitude between groups.

### 3.2 Diet Richness, Diversity, Evenness, and Overlap

Across study groups, a total of 226 food type‐items (representing a minimum of 109 different food types) were recorded via focal and group scan sampling (Table S3), and an additional 30 food type‐items were only recorded opportunistically (Table S4). A total of 57 food type‐items were newly added to the list of Virunga mountain gorilla foods (Table S5). During the study period, the number of consumed food type‐items, which refers to specific parts of food types (e.g., a plant species), varied between 17 and 29 in SW groups and between 90 and 131 in NE groups (Table S3).

All key foods (type‐items) that made up ~80% of each study group's diet were plant matter and differed greatly between SW and NE groups (Table 4), with only 8 out of 26 key foods being shared. NE groups relied on a larger number of key foods (10–17) compared to SW groups (5–8). In addition, lianas and trees/shrubs played a more important role in the diet of NE groups than in the diet of SW groups, which mostly relied on a few herbs. The preference for seasonally occurring bamboo shoots was shared across groups of both VNP regions, except for two SW groups (Titus and Ntambara), for which shoots were not part of the key foods during the study period. An unexpected result is that three exotic tree species planted adjacent to the park border and fed on during visits outside the protected area, including *Acacia melanoxylon* , *A. mearnsii* , and *Eucalyptus* spp., belong to the key foods of NE groups (Table 4).

**TABLE 4:** Diet composition of southwest (gray) and northeast (white) groups presented by key food type‐items (making up ~80% of diet), importance of each key food type‐item (%), and food type life form, observed diet richness (total number of food type‐items in a group's diet), observed and estimated diet diversity (Hill‐Shannon: *Q* = 1), Pielou index for diet evenness (0–1), and sampling effort (total feeding observations).

Food type (species)	Item	Form	Groups											
			DUS	PAB	MSK	KRB	TOB	TIT	SEG	NTA	SAB	MUH	KSA	KWI
Ferns														
Ferns	Stalk	Fern							2.1					1.1
*Pleopertis macrocarpa*	All	Fern											1.9	4.2
		**Total**	**0**	**0**	**0**	**0**	**0**	**0**	**2.1**	**0**	**0**	**0**	**1.9**	**5.3**
Grasses														
*Oldeania alpina*	Leaf	Grass									12.8	7.7	6.0	8.2
*Oldeania alpina*	Shoot	Grass	15.0	15.0	17.1	9.9	19.0		4.0		21.7	17.7	19.2	16.6
*Cyperus* spp.	Leaf	Grass					6.6							
		**Total**	**15.0**	**15.0**	**17.1**	**9.9**	**25.0**	**0**	**4.0**	**0**	**34.5**	**25.4**	**25.2**	**24.8**
Herbs														
*Thistles*	Leaf	Herb	17.0	19.0	23.2	35.0	24.0	14.7	23.0	17.0	6.3	7.3	2.7	2.9
*Thistles*	Root	Herb			6.6	4.1	4.5	8.8		4.3	3.0	6.0	2.8	1.9
*Thistles*	Stem	Herb		6.9				3.7	15.0	2.6				1.3
*Laportea alatipes*	Leaf	Herb	3.8			3.0		6.2	4.0	3.2				
*Afroligusticum linderi*	Stem	Herb	22.0	16.0	7.6	11.0	3.2	15.8	9.2	30.0				
*Galium spp*	All	Herb/liana	23.0	23.0	30.3	20.0	19.0	26.2	21.0	25.0	3.1	2.1	2.2	2.0
		**Total**	**6.05**	**65.0**	**67.7**	**73.0**	**50.0**	**75.4**	**72.2**	**82.0**	**12.4**	**15.4**	**7.7**	**8.1**
(Woody) lianas, shrubs, trees														
*Basella alba*	Leaf	Liana									5.1	8.2	4.9	3
*Clematis simensis*	Pith	Liana											4.7	7.7
*Droguetia iners*	Leaf	Liana									3.8			
*Secamone africana*	Leaf	Liana										5.7	5.8	9.1
*Englerina spp*	Stem	Shrub											2.0	2.0
*Rubus spp*	Leaf	Shrub					5.4		4.0				1.3	
*Acacia melanoxylon* ^*E*^	Bark	Tree												1.3
*Eucalyptus spp* ^*E*^	Bark	Tree									3.0		1.5	
*Eucalyptus spp* ^*E*^	Sap	Tree										7.7	8.7	
*Acacia mearnsii* ^*E*^	Leaf	Tree/Shrub											2.2	
*Acacia mearnsii* ^*E*^	Stem	Tree/Shrub											1.3	
*Discopodium penninervium*	Pith	Tree/Shrub						5.7						2.1
*Solanecio mannii*	Pith	Tree/Shrub												1.0
*Scepocarpus hypselodendron*	Bark	Woody liana									15.1	10.3	8.6	10.3
*Scepocarpus hypselodendron*	Leaf	Woody liana									6.2	9.2	5.2	5.7
		**Total**	**0**	**0**	**0**	**0**	**5.4**	**5.7**	**4.0**	**0**	**33.2**	**41.1**	**46.2**	**42.2**
**% key foods**			**80.0**	**80.0**	**84.7**	**83.0**	**81.0**	**81.2**	**82.0**	**82.0**	**80.1**	**81.9**	**80.8**	**80.4**
**N key food type‐items**			**5**	**5**	**5**	**6**	**7**	**7**	**8**	**6**	**10**	**10**	**17**	**17**
**Observed richness**			**21**	**19**	**17**	**22**	**22**	**39**	**33**	**31**	**93**	**105**	**112**	**128**
**Observed diversity**			**7.3**	**8.4**	**6.84**	**8**	**9.4**	**11.4**	**10.5**	**7.7**	**19.2**	**21.8**	**28.5**	**28.6**
**Estimated diversity**			**7.6**	**8.5**	**7.16**	**8.2**	**9.6**	**11.7**	**10.8**	**8.2**	**19.8**	**22.5**	**29.5**	**29.8**
**Estimated evenness**			**0.321**	**0.351**	**0.330**	**0.340**	**0.345**	**0.364**	**0.363**	**0.345**	**0.368**	**0.431**	**0.384**	**0.427**
**Total observations**			**655**	**548**	**380**	**558**	**895**	**828**	**996**	**585**	**2266**	**2284**	**2093**	**2368**

*Note:* Bolded values highlight the summary of key findings.

**FIGURE 5:**
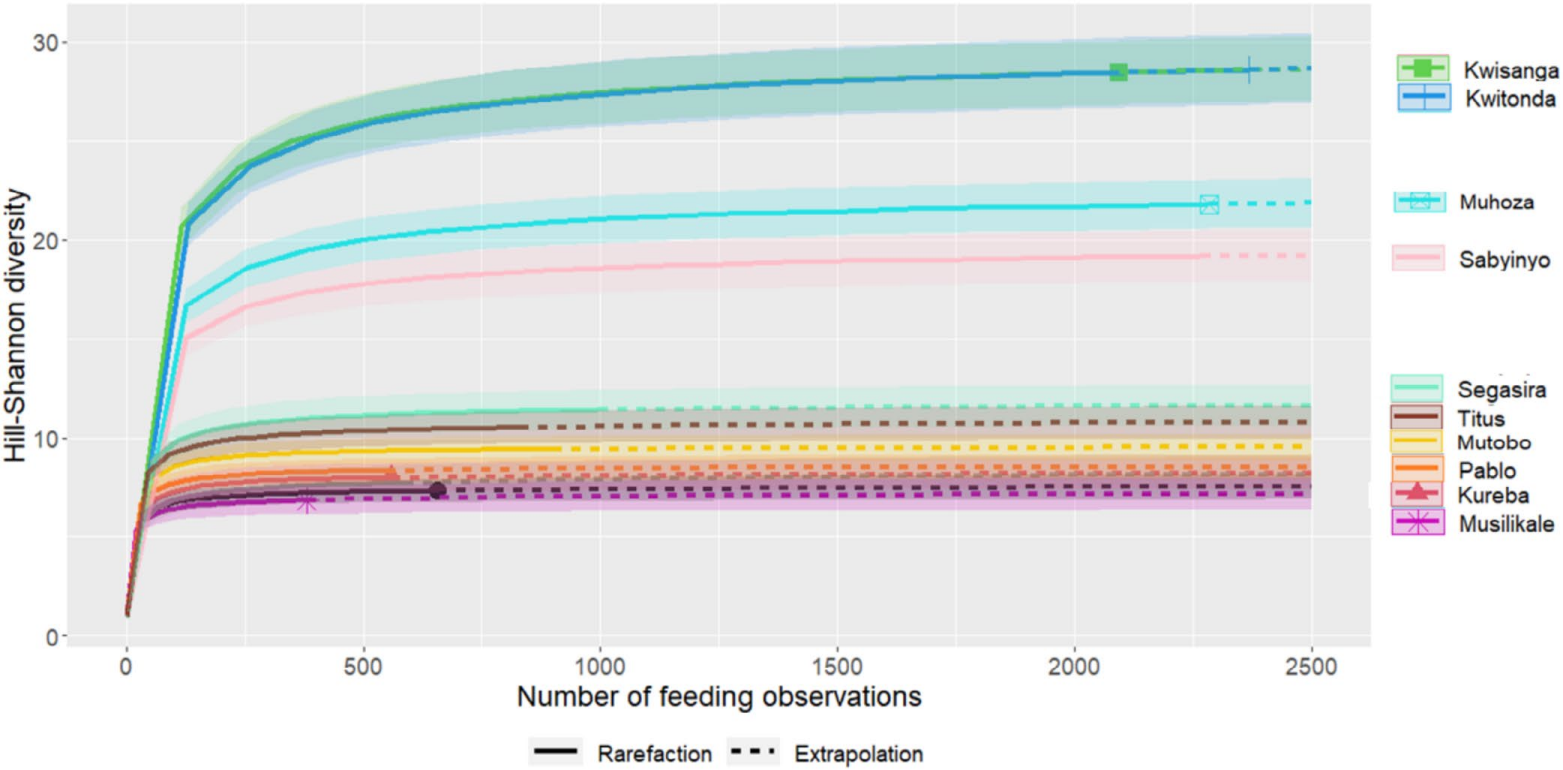
Rarefaction and extrapolation sampling curves for Hill‐Shannon diversity (*q* = 1) computed from feeding observations of food type‐items consumed (sample‐based) by study groups (northeast groups represented by the four upper curves: Kwisanga, Kwitonda, Mutobo, and Sabyinyo).

**FIGURE 6:**
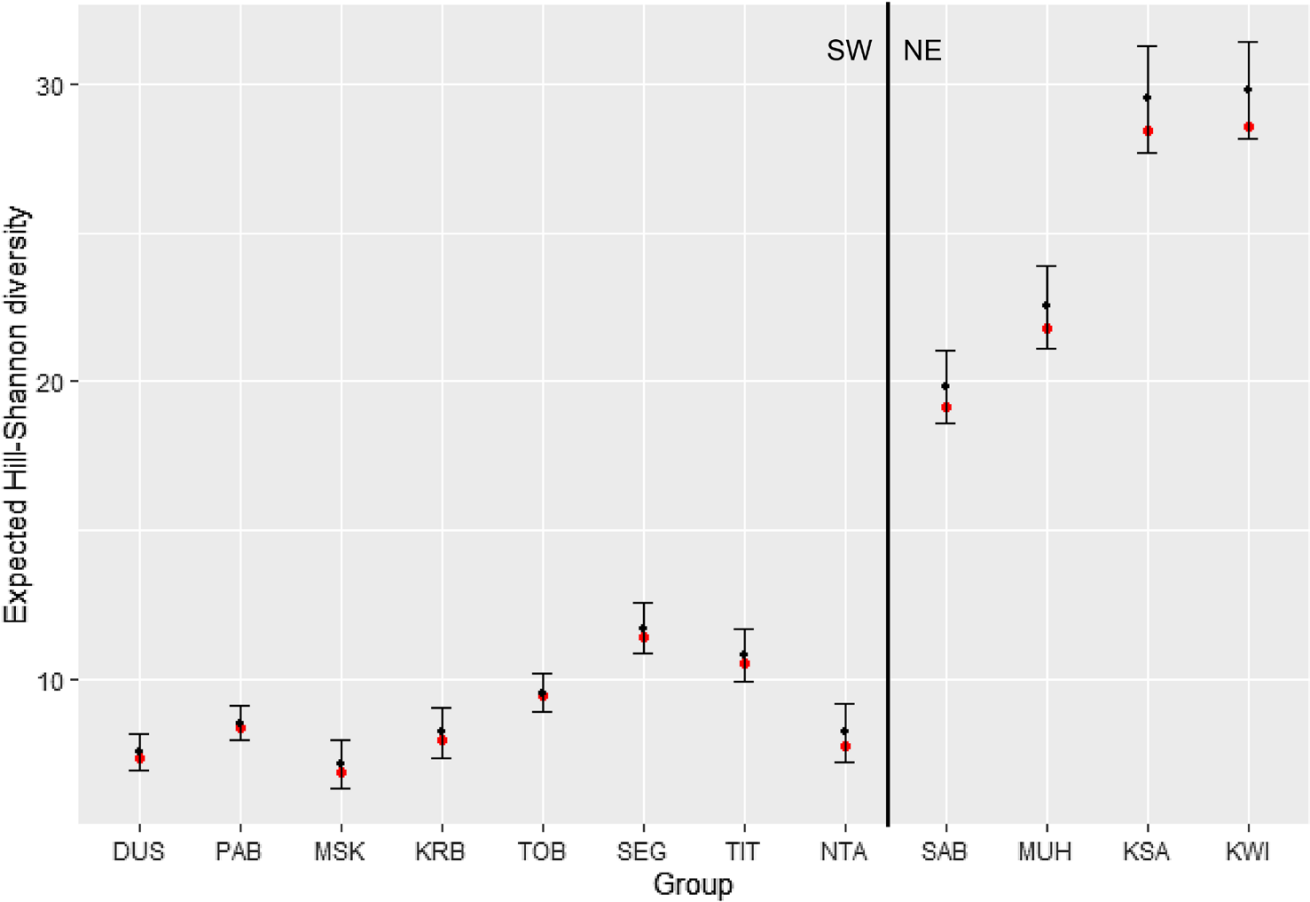
Observed (red dots) and expected (black dots; set at 2500 observations) diet diversity (Hill‐Shannon: *Q* = 1) with 95% upper and lower confidence limits by group (Northeast groups SAB, MUH, KSA, KWI; others belong to SW groups). Confidence limits between groups without overlap indicate distinct diet diversities.

**FIGURE 7:**
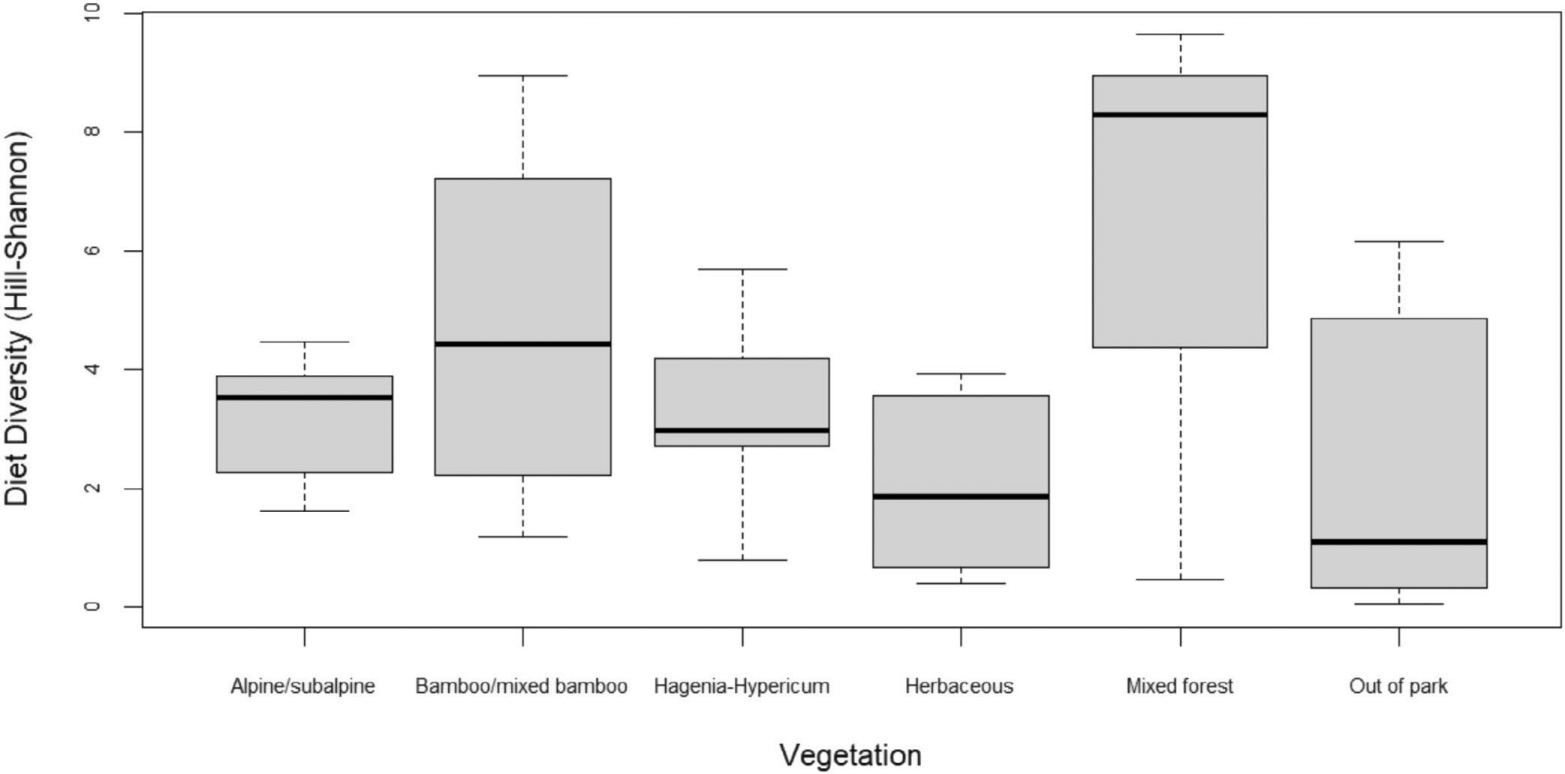
Between‐group variation in observed diet diversity (Hill‐Shannon: *Q* = 1) within vegetation types that were used by multiple groups.

Overall, the observed and expected diet diversities (Hill‐Shannon) values were substantially higher in NE groups than in SW groups (Table 4, Figures 5 and 6) with the most diverse diet associated with Kwitonda and Kwisanga groups. These groups differed from all other groups by ranging in the “mixed forest” zone in the easternmost VNP region. Musilikale and Dushishoze groups, which ranged in the southernmost region and used the “alpine/subalpine zone” for extended periods of the study, had the lowest diet diversity. We also found strong variation in diet diversity among groups within the same vegetation zone (Figure 7, Tables S6–S13). Because the four NE groups spent substantial time in the “bamboo/mixed bamboo zone” throughout the year independent of shoot presence, unlike SW groups, we also calculated the importance of food type‐items in this zone on days NE groups did not feed on bamboo shoots (Table S12). In those days, their most important foods were *Scepocarpus hypselodendron* bark and bamboo leaves. Although all groups had relatively unbalanced diets, indicated by a low diet evenness, slightly higher diet evenness was linked to NE groups.

The described dietary difference resulted in high diet overlap within SW groups (0.790–0.932) and within NE groups (0.854–0.941) but only low to medium diet overlaps between groups in the different regions (0.165–0.550) (Table 5).

**TABLE 5:** Diet overlap (Pianka's index) matrix of southwest (SW) and northeast (NE) groups (gray indicates overlap between both subpopulations).

	SW							NE			
	PAB	MSK	KRB	TOB	SEG	TIT	NTA	SAB	MUH	KSA	KWI
DUS	0.971	0.897	0.862	0.822	0.826	0.891	0.906	0.421	0.414	0.392	0.351
PAB		0.932	0.903	0.878	0.844	0.930	0.866	0.446	0.432	0.406	0.364
MSK			0.908	0.940	0.906	0.879	0.769	0.465	0.471	0.425	0.380
KRB				0.907	0.916	0.831	0.790	0.392	0.417	0.316	0.295
TOB					0.845	0.878	0.638	0.548	0.550	0.500	0.452
SEG						0.923	0.823	0.323	0.377	0.260	0.238
TIT							0.930	0.271	0.297	0.229	0.218
NTA								0.165	0.187	0.127	0.120
SAB									0.918	0.887	0.854
MUH										0.941	0.855
KSA											0.900

### 3.3 Diet Quality

All key foods of a study group's diet (Table 4) were sampled for nutrient assessment (see Table S2), except for leaves and stems of *Acacia mearnsii* consumed by the Kwisanga group. The group started feeding on this tree after a drastic home range shift following the group split from the Kwitonda group in May 2021. We therefore exclude the Kwisanga group from the following analyses.

**FIGURE 8:**
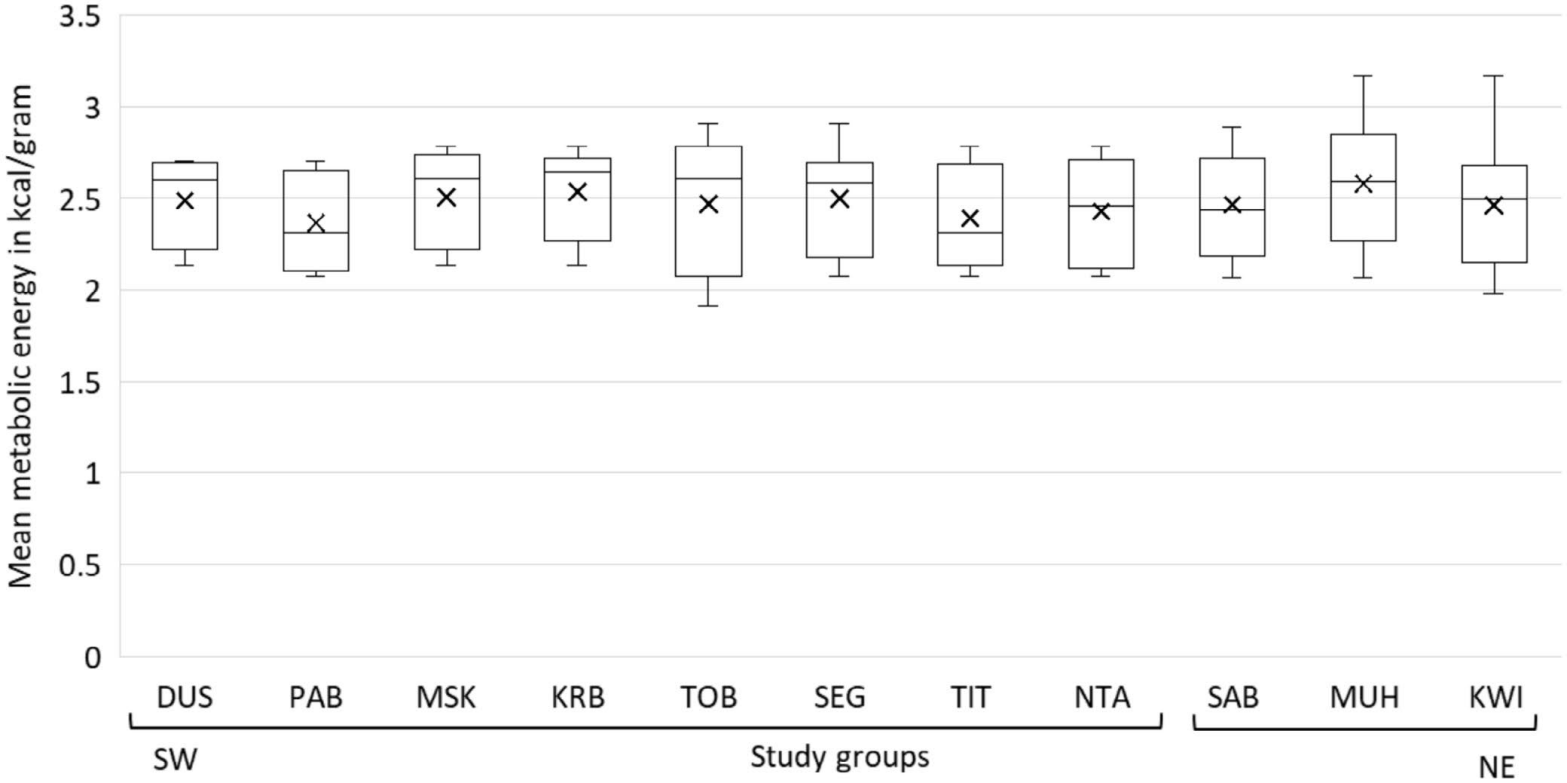
Boxplots showing median, mean (cross symbol), interquartile range, and min/max of metabolic energy in kcal/g in key food type‐items making up 80% of the diet by study group in the southwest (SW) and the northeast (NE) of the park. Note that foods included in the diet of KSA only make up 77.5% because *Acacia mearnsii* was not sampled for nutritional analysis.

**FIGURE 9:**
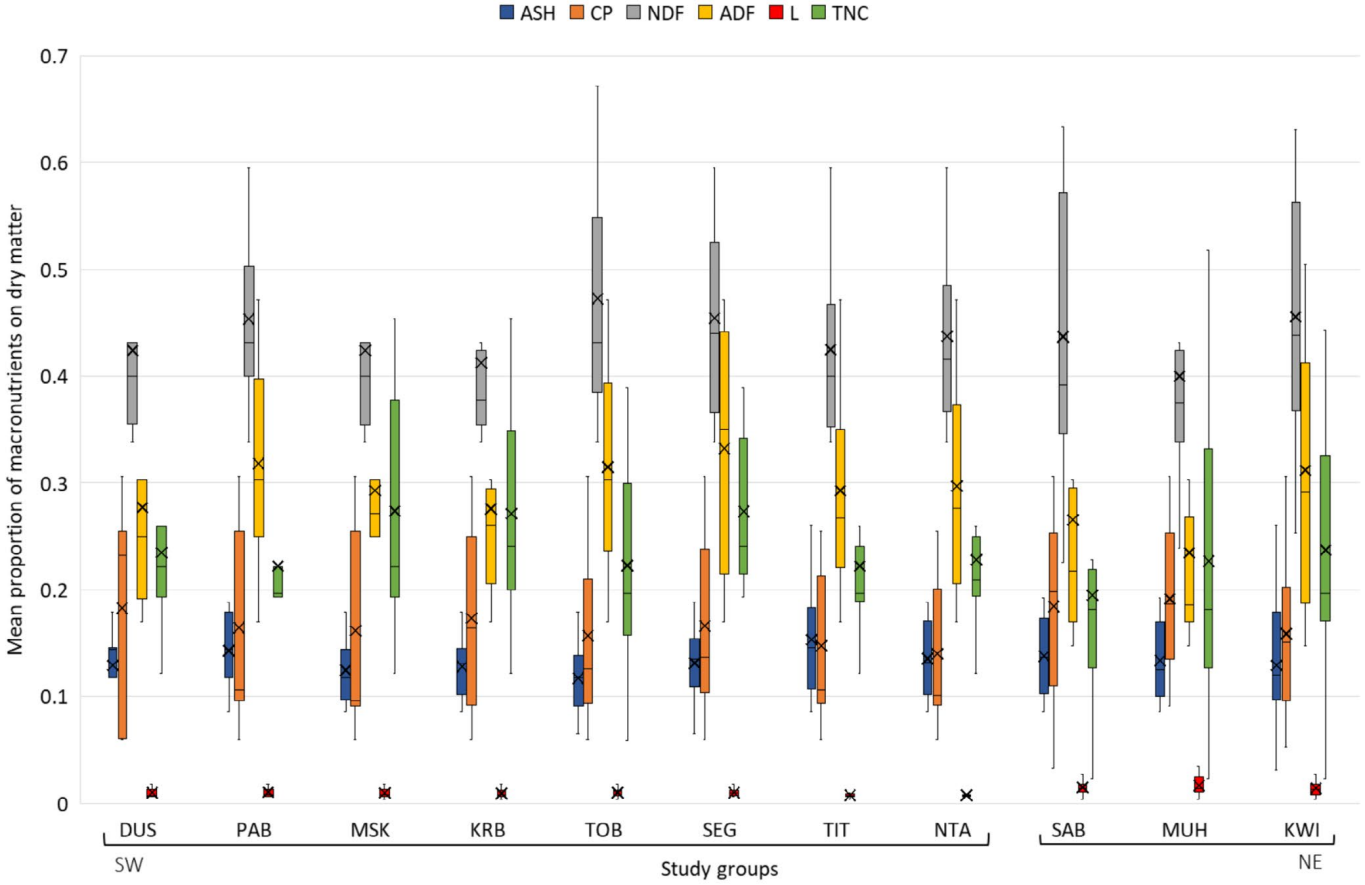
Boxplots showing median, mean (cross symbol), interquartile range, and min/max of the mean proportion of macronutrients (ADF, acid detergent fiber; ASH, total ash; CP, crude protein; L, lipids; NDF, neutral detergent fiber; TNC, total nonstructural carbohydrates) in key food type items making up 80% of the diet by study groups in the Southwest (SW) and Northeast (NE) of the park. Note that foods included in the diet of KSA only make up 77.5% because *Acacia mearnsii* was not sampled for nutritional analysis.

In our first analysis, we ignored the importance of each food in the diet of groups and found no between‐group difference in the mean metabolic energy of key foods (Kruskal–Wallis test: *H* = 2.907, df = 10, *p* = 0.984), ASH (GLM: *D* = 1.411, *p* = 0.943), CP (GLM: *D* = 4.238, *p* = 0.961), NDF (GLM: *D* = 3.970, *p* = 0.996), ADF (GLM: *D* = 4.786, *p* = 0.953), and TNC (GLM: *D* = 6.154, *p* = 0.998) (Figures 8 and 9). However, we found a significant group effect on the proportion of lipids in key foods (*D* = 0.307, *p* = 0.046), with those of four SW groups (KRB, SEG, TIT, NTA) having lower mean lipid proportions per gram dry matter compared to Muhoza (NE group) (Table S14), which had the highest mean lipid proportion per gram dry matter in key foods (0.017) (Figure 9). Key foods of NE groups with relatively high mean lipid proportions that were not consumed by SW groups and/or that were of lower importance in their diet include leaves of *Secamone africana* (0.035), *Oldeania alpina* (0.028), *Basella alba* (0.026), and *Scepocarpus hypselodendron* (0.035).

Next, we weighted the mean proportions of macronutrients and mean metabolic energy (kcal/g) of key foods (covering 80% of their diet) in each group's diet by their dietary importance (percentage of feeding time) and calculated the sum of these weighted values (Table 6, Figures S1, S2). Key foods of NE groups had higher weighted mean proportions of L than key foods of SW groups. No difference in the diet by group location was found for weighted proportions of ASH, NDF, ADF, and TNC, as well as for MEC.

**TABLE 6:** *T*‐test statistics comparing mean weighted proportions of macronutrients and mean metabolic energy concentrations of key foods (covering 80% of their diet) of southwest (SW) groups (*N* = 8) and northeast (NE) groups (*N* = 3, Kwisanga was excluded).

Dataset	Mean ± SD SW	Mean ± SD NE	*t*	*p*
Metabolic energy (MEC)	194.13 ± 8.26	199.89 ± 5.43	1.343	0.230
Crude protein (CP)	14.99 ± 2.12	16.79 ± 1.33	1.673	0.145
Lipids (L)	0.89 ± 0.11	1.39 ± 0.01	12.140	**< 0.001**
Inorganic matter (ASH)	11.92 ± 0.48	10.92 ± 0.83	−1.957	0.162
Neutral detergent fiber (NDF)^a^	24.55 ± 2.06	21.35 ± 2.29	11.000	0.921
Acid detergent fiber (ADF)	36.16 ± 1.34	34.84 ± 2.39	−0.903	0.445
Total nonstructural carbohydrates (TNC)	17.08 ± 0.83	16.12 ± 2.56	−0.633	0.587

*Note:* Bold indicates a level of significance  <  0.05.

^a^ ran Mann–Whitney *U* test and retrieved W‐statistic because data were not normal distributed.

## 4 Discussion

### 4.1 Dietary Pattern

This first snapshot capturing dietary profiles of mountain gorilla groups across the Volcanoes National Park (VNP) in Rwanda confirms considerable dietary flexibility of this endangered ape population as predicted and previously demonstrated within a restricted VNP region (McNeilage 1995, 2001; Watts 1984). The diet of the NE study groups ranging in lower elevations is more diverse (86–123 observed food type‐items) and contains a larger number of key foods (10–17) that make up ~80% of their feeding time compared to the diet of the SW study groups ranging at higher elevations (18–43 observed food type‐items including 5–8 key foods). In addition, the plant species and food type‐item composition of key foods strongly differs between both subpopulations with little to medium overall diet overlap among SW and NE groups (16.5%–55%), which is lower overlap than previously reported between mountain gorillas and other large mammal species residing in the SW region of the VNP (Plumptre 1995). Studying VNP groups within a subset of the forest area of the SW groups, Watts (1984) previously showed that spatial rather than temporal variability in the diet is associated with the composition of foods available in distinct vegetation zones. Watts also detected an inverse relationship between dietary evenness and absolute food biomass per vegetation zone. If this relationship extends to the NE groups and their home ranges, we expect that this understudied forest area holds lower absolute food biomass than the historical study site in the southwest region, where groups show lower evenness in their diet than NE groups. Future studies need to investigate the relations between food distribution, diversity, and biomass in the east of the VNP. This study has laid an important foundation for this future research by identifying foods consumed by groups in this area. Dietary differences linked to variation in habitat types and elevation were also documented in the mountain gorilla population living in the Bwindi Impenetrable National Park (Bwindi), which relies largely on fibrous foods like the Virunga population but incorporates more fruits into the diet (Ganas et al. 2004). Temporal and spatial differences in food availability were suggested to be responsible for the pattern in dietary diversity among Bwindi groups.

Noticeable variability in diet diversity between VNP groups in the SW and NE was also found within major vegetation zones, mirroring outcomes from an earlier study restricted to the Karisoke research areas and its typical vegetation zones, excluding the bamboo zone (Watts 1984). Our feeding data obtained from the “bamboo/mixed bamboo” zone revealed that the diet diversity of NE groups in the lower elevations of this zone exceeded the values of the SW groups (Table S8), which use higher elevations of this zone. The set of abiotic and biotic parameters that determines plant compositions and communities (Belyea and Lancaster 1999) changes along the elevational gradient providing conditions for the formation of microhabitats within zones. This finding also suggests that many food type‐items are yet missing from the updated food list of this mountain gorilla population. For example, the “mixed forest” zone on the DRC and Uganda side of the Virunga Massif reaches much lower elevations (~2000 m) than the small remaining “mixed forest” patches in Rwanda (~2400 m), but the feeding ecology of groups in these neighboring countries has not been studied. Furthermore, spatial differences in plant composition within vegetation zones in the Virunga Massif require more research considering their potentially significant implications for animal ecology. Endangered golden monkeys ( *Cercopithecus mitis kandti* ), the only other primates in the VNP, are predominantly found in the “bamboo/mixed bamboo zone” and provide an example (Tuyisingize et al. 2022b); groups only 16 km apart adapted two different birthing seasons linked to the highest availability of bamboo shoots, which coincides with the heavy rainy season at high elevations and with the short rainy season at low elevations. Future remote‐sensing studies will be important to characterize fine‐scale spatial–temporal properties of macro‐ and microhabitats within the VNP and will enable monitoring of changes over time. This kind of more comprehensive knowledge of habitats and structural diversity of the Virunga Massif will be necessary to understand spatial variation in the population dynamics of these endangered primate species.

### 4.2 Diet Quality of Key Foods

We also tested whether NE groups rely on key foods characterized by lower nutritional quality than SE groups, as one possible factor responsible for previously slower population growth rates in the eastern region of the VNP. However, this first glimpse into the nutritional composition of key foods within this population does not support this hypothesis. Instead, our findings are in line with two comparative studies, between both mountain gorilla populations (Rothman et al. 2007) and between different western lowland gorilla ( *Gorilla gorilla gorilla* ) populations (Robbins et al. 2022), which reported remarkable similarities in the nutritional concentrations of their diet despite occupying different habitats. Nutritional concentrations of foods measured in this study are also comparable to and largely within the range of those available from previous analyses of mountain gorilla foods (Rothman et al. 2006, 2007) and foods of western lowland gorillas (Calvert 1985; Lodwick and Salmi 2019; Popovich et al. 1997; Rogers et al. 1990) (Table S15). Contrary to our prediction, key foods of Muhoza group in the eastern region had significantly higher lipid contents than four of the eight SW groups, though lipid values were still quite low and contributed only a small proportion to the calculated metabolic energy. Weighted nutritional values of key foods (taking into account their importance in the diet) also suggest an advantage in obtaining lipids from key foods for NE groups over SW groups. Whether these statistical differences are of biological relevance is difficult to interpret considering the overall small proportional differences in lipid among key foods across study groups (0.3%–3.5%) and the observation that none of the differences translated into detectable differences in the metabolic energy of key foods.

However, we cannot yet exclude that variation in the nutritional quality of the diet between both subpopulations (SW and NE groups) contributed to heterogeneous growth rates in the VNP for various reasons that require follow‐up research. First, we weighed nutritional values by the importance of foods in the diet based on time spent feeding on each food, which does not necessarily correlate with actual food intake. Second, substantial spatial and temporal variability in nutritional composition can occur within plant species and even in an individual plant and its parts (Rothman et al. 2012, 2014). Third, less frequently consumed foods, which were not examined for nutrition in this study, may also be of great importance for maintaining health by providing crucial micronutrients such as sodium (Rothman et al. 2006; Grueter et al. 2018) or other important compounds with medicinal value (Huffman 2001, 2017) and thus should not be ignored. Although we cannot be certain, methodological differences between labs in reporting NDF and ADF values (with or without residual ash) unlikely contributed to the overall lack of qualitative differences in NDF, ADF, and resulting TNC and metabolic energy values between foods of both subpopulations because we calculated mean nutritional values across sampling periods for each food, and test statistics for these components were far from reaching the significance level.

A note of caution needs to be applied to our calculated metabolizable energy values for the plant foods assayed in this study. Total nonstructural carbohydrates (TNC) were the second largest component of the equation for metabolic energy (next to NDF) in terms of quantity. TNC is difficult to measure directly with considerable variability in results from different laboratories (Quentin et al. 2015). We calculated TNC by difference. The TNC fraction of these plant foods likely has a high proportion of digestible carbohydrates, such as simple sugars and starches (Milton 2008), which would provide gorillas the proposed 4 kcal/g when ingested. However, the TNC fraction also can include oligosaccharides (e.g., raffinose), fructans (e.g., inulin), and sugar alcohols (e.g., mannitol) (Stick and Williams 2010), which are resistant to mammalian endogenous digestive enzymes and would need to be fermented in the hindgut. In addition, tannins and other plant secondary compounds were not measured in this study and, as such, would also be “included” in the TNC fraction. Thus, our assumed value of 4 kcal/g for TNC is likely an overestimate.

### 4.3 Alternative Explanations for Heterogeneous Gorilla Population Growth in the VNP

Our preliminary findings on key food quality highlight the importance of considering additional explanations for spatial differences in gorilla population growth in the VNP for over two decades. If food nutritional compositions and daily energetic intakes are relatively uniform among VNP groups as shown across mountain gorilla populations (Rothman et al. 2007; Wright et al. 2015), those located in the eastern region with historically slower growth rates may experience higher energetic costs in acquiring foods for meeting daily metabolic requirements to an extent that slows development and reduces reproductive success. For example, foods in the eastern region may be less evenly distributed and/or available at lower density or biomass, leading to longer daily travel distances (Carbone et al. 2005; Isbell 1991; Raño et al. 2016; Wright et al. 2015). In addition, frequent vertical climbing of trees to harvest vines, which make up large portions of the eastern groups' diet, may require more energy than harvesting terrestrial herbaceous vegetation (Pontzer and Wrangham 2004), which is strongly preferred by SW groups.

Alternatively, slow population growth in the most eastern section of the VNP could be a phenomenon known as the Allee effect (Angulo et al. 2018), which predicts low population growth at low population density if aggregation of conspecifics is beneficial. Long‐term demographic and ranging data from the mountain gorilla subpopulation in the Karisoke research area showed that lower group densities are associated with smaller home range overlaps and fewer intergroup encounters between neighboring groups (Caillaud et al. 2020). Although intergroup encounters can cause lethal injuries in mature males and infants, they are important opportunities for females to transfer between groups, a strategy to avoid inbreeding and increase the survival of future offspring (Caillaud et al. 2020; Morrison et al. 2023; Robbins and Robbins 2018). However, if groups range largely isolated from other social groups, intergroup encounters and female dispersal may become extremely rare or even cease and slow local population growth. Due to limited gene flow between groups, more isolated groups may also face a higher risk of inbreeding, which can negatively affect development, fertility, health, and survival as widely documented in humans and other animals (Charlesworth and Charlesworth 1987; Charpentier et al. 2007; Fareed and Afzal 2017; Postma et al. 2010), and thus further hinder local population growth. Apart from the large savannah‐like grasslands and bare fields of lava rocks on the northeast slopes of Mount Muhabura, both intensive fires in this area in 1989 (surface area is unknown) and 2009 (~300 ha) likely created additional barriers, which further hampered encounters between groups ranging on the northern slopes in Uganda and southern slopes in Rwanda. Finally, we may find that the answer to understanding heterogeneous population growth rates in the VNP is more complex and instead reflects a combination of causal factors.

### 4.4 Conservation Implications and Management

Regular monitoring of bamboo shoot, the only key gorilla food that is consumed by groups across the VNP, has revealed a recent decline in bamboo shoot regeneration (van der Hoek et al. 2019). If this trend continues, NE groups will likely be more impacted by a reduced availability of this temporal food source that is rich in protein and fiber (Grueter et al. 2016), with potential negative effects on fitness and reproduction. Eventually, current forest areas characterized as “bamboo/mixed bamboo zone” might be transformed into other vegetation zones, forcing NE groups to adapt to new dietary compositions. Another result from this study requires more attention in future monitoring and conservation efforts. The NE groups incorporated three exotic tree species found outside the park into their key foods. Although SW groups also feed on *Eucalyptus* spp., they do so in much lower proportions (Table 4, Table S3). Ranging year‐round at lower elevations near the park boundaries may attract these groups to foods outside the protected areas more frequently than the SW groups. During recent visits outside the park, Kwitonda also discovered two other exotic fruits, tree tomato ( *Solanum betaceum* ) and papaya ( *Vasconcellea pubescens* , syn. *Carica cundinamarcensis*) (Table S3), planted in the nearby fields. These new food sources are rich in a wide range of nutrients (Da Silva et al. 2007; Wang and Zhu 2020). They could attract groups more frequently to outside‐park areas in the future, which may increase their exposure to infectious diseases from livestock and humans (Hogan et al. 2014), chemicals such as fertilizers and insecticides, and in the longer term provide these exotic plants a pathway to spread in forest areas through seeds being dispersed with gorilla feces after returning to their natural habitat.

Our findings add to existing primate literature stressing the implications of overgeneralizing a species' diet variability for models projecting species distribution and survival and on conservation actions (e.g., Ganas et al. 2004 for Bwindi mountain gorillas). In the context of mountain gorilla conservation, they are also most timely. The updated list of mountain gorilla food plants and advanced understanding of spatial variability in diet profiles across the VNP will aid the planning of park restoration efforts initiated by the Rwandan government. In light of climate change, important gorilla food plants in the lower elevational range of the VNP today will likely be more resilient under future climate scenarios in the restoration areas at elevations below the current park border. Furthermore, existing information about the diet and habitats from the Bwindi gorilla populations (Ganas et al. 2004, 2008) covering elevations below the current VNP border is also of great value for restoration planning, considering the general trend of an upward shift of plant species with increasing temperature (Lenoir et al. 2008) coupled with the lack of feeding data from gorilla groups ranging in the lowest elevations of the Virunga Massif in DRC and Uganda.

## Author Contributions

**H. Ihimbazwe:** data curation (lead), formal analysis (lead), methodology (equal), visualization (lead), writing – original draft (lead), writing – review and editing (equal). **J. D. Tuyizere:** data curation (lead), methodology (equal), visualization (supporting), writing – review and editing (equal). **L. Kayitete:** data curation (lead), methodology (equal), writing – review and editing (equal). **D. Abavandimwe:** data curation (equal), methodology (equal), project administration (lead). **A. K. Kamanzi Shimwa:** data curation (lead), methodology (equal), resources (equal), writing – review and editing (equal). **M. L. Power:** data curation (equal), methodology (equal), writing – review and editing (equal). **C. C. Grueter:** data curation (equal), methodology (equal), resources (equal), writing – review and editing (equal). **M. Flint:** methodology (equal), writing – review and editing (equal). **J. D. Nsanzineza:** data curation (equal), writing – review and editing (equal). **A. Jonas:** data curation (supporting), writing – review and editing (equal). **G. Kwibuka:** data curation (supporting), writing – review and editing (equal). **D. Ishimwe:** data curation (equal), resources (lead). **F. Ndagijimana:** project administration (lead), writing – review and editing (equal). **J. D. Hakizimana:** project administration (lead), writing – review and editing (equal). **P. Uwingeli:** project administration (lead), writing – review and editing (equal). **S. C. McFarlin:** methodology (equal), project administration (lead), writing – review and editing (equal). **M. M. Robbins:** writing – review and editing (equal). **T. S. Stoinski:** conceptualization (lead), methodology (lead), project administration (lead), writing – review and editing (equal). **W. Eckardt:** conceptualization (lead), data curation (equal), formal analysis (lead), funding acquisition (equal), methodology (lead), project administration (lead), supervision (lead), visualization (lead), writing – original draft (equal), writing – review and editing (lead).

## Conflicts of Interest

The authors declare no conflicts of interest.

## Supporting information


Appendix S1.

Appendix S2.

## Data Availability

The data that support the findings of this study will be openly available at Dryad after the submission of the manuscript as soon as a manuscript number has been aligned. Nutritional data we used for the research analysis involved biological samples (plant specimens). The government of Rwanda through the Rwanda Development Board (RDB) requires that publications resulting from biological samples collected within Rwanda need to be first approved by RDB before submission/publication. In addition, biological samples used for a specific research project approved by RDB cannot be used for other research projects before approval by RDB. Due to these regulations, the datasets cannot be made publicly available on a depository platform but can be requested through the Dian Fossey Gorilla Fund and shared upon approval by the Rwanda Development Board.
