# Supplementary material for: Dietary Variability Among Mountain Gorilla Groups Across Volcanoes National Park, Rwanda
Source: Ecol Evol. 2025 May 15;15(5):e71192. doi: 10.1002/ece3.71192 (PMC12081832; doi:10.1002/ece3.71192)
Supplement: Supplementary file 1 — Appendix S1. [file ECE3-15-e71192-s001.docx]

Supplementary Table 1. Summary of GPS coordinates of group locations (N nest and noon) collected during the study period between January 2020 and December 2022 (start indicates when data collection was initiated in the group) and used for data analysis. Grey indicating southwest groups and white indicating northeast groups.

| Group | N nest | N noon | Total N | N days | Start |
| --- | --- | --- | --- | --- | --- |
| DUS | 301 | 539 | 840 | 370 | Apr 2021 |
| PAB | 470 | 942 | 1412 | 989 | Jan 2020 |
| MSK | 359 | 870 | 1229 | 916 | Jan 2020 |
| KRB | 434 | 906 | 1340 | 965 | Jan 2020 |
| TOB | 582 | 948 | 1530 | 1015 | Jan 2020 |
| SEG | 114 | 225 | 339 | 226 | May 2022 |
| TIT | 556 | 999 | 1555 | 1030 | Jan 2020 |
| NTA | 472 | 927 | 1399 | 975 | Jan 2020 |
| SAB | 208 | 619 | 827 | 685 | Jan 2020 |
| MUH | 419 | 744 | 1163 | 847 | Jan 2020 |
| KSA | 162 | 435 | 597 | 461 | Aug 2021 |
| KWI | 420 | 746 | 1166 | 851 | Jan 2020 |

Supplementary Table 2. Number of collected key food plant species-item included in data analysis by sampling period (P1-5).

| **Key food plant species** | **Part** | **P1** | **P2** | **P3** | **P4** | **P5** | **Total** |
| --- | --- | --- | --- | --- | --- | --- | --- |
| *Acacia melanoxylon* | Bark | - | - | - | - | 5 | **5** |
| *Afroligusticum linderi* | Stem | 1 | 1 | 3 | 8 | 2 | **15** |
| *Basella alba* | Leaf | 1 | - | - | - | 4 | **5** |
| *Clematis simensis* | Pith | - | - | - | - | 4 | **4** |
| *Cyperus spp.* | Leaf | 1 | - | - | - | 1 | **2** |
| *Discopodium penninervium* | Pith | 1 | - | - | - | 4 | **5** |
| *Droguetia iners* | Leaf | 1 | - | - | 6 | 3 | **10** |
| *Englerina spp* | Stem | - | - | - | - | 1 | **1** |
| *Eucalyptus spp* | Bark | 1 | - | - | - | 2 | **3** |
| *Eucalyptus spp* | Sap | - | - | - | - | 2 | **2** |
| Ferns | Stalk | - | - | - | - | 1 | **1** |
| *Galium spp* | All | 2 | 1 | 9 | 11 | 4 | **27** |
| *Laportea alatipes* | Leaf | 1 | 1 | - | 7 | 1 | **10** |
| *Oldeania alpina* | Leaf | 1 | - | - | - | 4 | **5** |
| *Oldeania alpina* | Shoot | 1 | 2 | - | - | 2 | **5** |
| *Pleopertis macrocarpa* | All | - | - | - | - | 2 | **2** |
| *Rubus spp* | Leaf | 2 | 1 | 6 | 6 | 3 | **18** |
| *Scepocarpus hypselodendron* | Bark | - | - | - | - | 4 | **4** |
| *Scepocarpus hypselodendron* | Leaf | - | - | - | - | 4 | **4** |
| *Secamone africana* | Leaf | - | - | - | - | 4 | **4** |
| *Solanecio mannii* | Pith | - | - | - | - | 4 | **4** |
| Thistle | Leaf | 3 | 2 | - | 9 | 9 | **23** |
| Thistle | Root | 1 | 2 | - | 8 | 7 | **18** |
| Thistle | Stem | 1 | 2 | - | 6 | 6 | **15** |
| Total |  | **18** | **12** | **18** | **61** | **83** | **192** |

^*^Nomenclature has changed (find previously accepted names in Supplementary Table 5)

Supplementary Table 3. Importance (in % of feeding observations) of food type-items in the diet of the 12 study groups (southwest groups in grey, northeast groups in white) sorted from left to right by their location in the Volcanoes National Park from the furthest southwest (DUS=Dushishoze) to the furthest east (KWI=Kwitonda).

| ***Food item*** | **Part** | **Family** | **DUS** | **PAB** | **MSK** | **KRB** | **TOB** | **TIT** | **SEG** | **NTA** | **SAB** | **MUH** | **KSA** | **KWI** |
| --- | --- | --- | --- | --- | --- | --- | --- | --- | --- | --- | --- | --- | --- | --- |
| ***Acacia mearnsii*** *^N^* | Leaf | Fabaceae |  |  |  |  |  |  |  |  |  | 0.57 | 2.2 |  |
| ***Acacia mearnsii*** *^N^* | Bark | Fabaceae |  |  |  |  |  |  |  |  |  | 0.04 | 0.1 | 0.04 |
| ***Acacia mearnsii*** *^N^* | Dry bark | Fabaceae |  |  |  |  |  |  |  |  |  |  | 0.05 |  |
| ***Acacia mearnsii*** *^N^* | Flower | Fabaceae |  |  |  |  |  |  |  |  |  |  | 0.1 |  |
| ***Acacia mearnsii*** *^N^* | Stem | Fabaceae |  |  |  |  |  |  |  |  |  | 0.35 | 1.34 | 0.04 |
| ***Acacia melanoxylon*** *^N^* | Bark | Fabaceae |  |  |  |  |  |  |  |  | 0.04 |  | 0.19 | 1.27 |
| ***Acacia melanoxylon*** *^N^* | Dry bark | Fabaceae |  |  |  |  |  |  |  |  |  |  |  | 0.3 |
| ***Acacia melanoxylon*** *^N^* | Deadwood | Fabaceae |  |  |  |  |  |  |  |  |  |  | 0.1 | 0.42 |
| ***Acacia melanoxylon*** *^N^* | Root | Fabaceae |  |  |  |  |  |  |  |  |  |  |  | 0.25 |
| ***Acacia melanoxylon*** *^N^* | Stem | Fabaceae |  |  |  |  |  |  |  |  |  |  |  | 1.01 |
| ***Afroligusticum linderi*** | Root | Apiaceae |  |  |  |  |  | 0.2 |  |  |  |  | 0.05 |  |
| ***Afroligusticum linderi*** | Stem | Apiaceae | 21.5 | 16.2 | 7.6 | 11.1 | 3.2 | 15.8 | 9.2 | 29.6 | 0.31 | 0.57 | 0.67 | 0.38 |
| ***Afroligusticum linderi*** | Leaf | Apiaceae |  |  | 0.3 |  |  |  |  |  |  |  |  |  |
| ***Afrosciadium kerstenii*** | Pith | Apiaceae |  |  |  |  |  |  |  | 0.2 |  |  |  |  |
| ***Afrosciadium kerstenii*** | Stem | Apiaceae |  |  |  |  |  |  |  | 0.2 |  |  |  |  |
| ***Ants*** | All |  |  |  |  |  |  |  |  |  |  | 0.22 |  | 0.04 |
| ***Baccharoides calvoana subsp. adolfi-friderici*** | Pith | Asteraceae |  |  |  | 0.2 | 0.4 | 0.1 | 0.2 |  |  |  | 0.1 | 0.3 |
| ***Baccharoides calvoana subsp. adolfi-friderici*** | Flower | Asteraceae |  |  |  |  |  |  |  | 0.7 |  |  |  |  |
| ***Basella alba*** | Flower | Basellaceae |  |  |  |  |  |  |  | 0.2 | 0.04 |  |  | 0.04 |
| ***Basella alba*** | Leaf | Basellaceae |  |  |  |  |  | 1.1 | 0.1 | 0.2 | 5.12 | 8.23 | 4.87 | 2.96 |
| ***Basella alba*** | Stem | Basellaceae |  |  |  |  |  |  |  |  | 0.04 | 0.09 | 0.24 | 0.17 |
| ***Begonia meyeri-johannis Engl*** *^N^* | Flower | Begoniaceae |  |  |  |  |  |  |  |  |  |  | 0.05 |  |
| ***Begonia meyeri-johannis Engl*** *^N^* | Stem | Begoniaceae |  |  |  |  |  |  |  |  |  | 0.04 |  |  |
| ***Bersama abyssinica*** *^N^* | Stem | Melianthaceae |  |  |  |  |  |  |  |  |  |  | 0.05 |  |
| ***Boehmeria cylindrica*** *^N^* | Leaf | Urticaceae |  |  |  |  |  |  |  |  | 0.18 |  | 0.05 | 0.13 |
| ***Boehmeria macrocalyx*** *^N^* | Bark | Urticaceae |  |  |  |  |  |  |  |  |  |  |  | 0.08 |
| ***Boehmeria macrocalyx*** *^N^* | Leaf | Urticaceae |  |  |  |  |  |  |  |  |  |  |  | 0.08 |
| ***Buddleja spp*** *^N^* | Leaf | Scrophulariaceae |  |  |  |  |  |  |  |  | 0.13 |  |  | 0.04 |
| ***Carex bequaertii*** | Leaf | Cypeaceae |  |  |  |  |  |  |  | 0.2 |  |  |  |  |
| ***Clematis simensis*** | Bark | Ranunculceae |  |  |  |  |  |  |  |  | 0.31 | 0.13 |  | 0.25 |
| ***Clematis simensis*** | Flower | Ranunculceae |  |  |  |  |  |  |  |  |  |  | 0.05 |  |
| ***Clematis simensis*** | Leaf | Ranunculceae |  |  |  |  |  |  |  |  |  |  | 0.1 | 0.04 |
| ***Clematis simensis*** | Pith | Ranunculceae |  |  |  |  |  |  |  |  | 0.35 | 1.23 | 4.73 | 7.73 |
| ***Clematis simensis*** | Root | Ranunculceae |  |  |  |  |  |  |  |  | 0.04 |  |  |  |
| ***Clematis simensis*** | Stem | Ranunculceae |  |  |  |  |  |  |  |  |  | 0.04 | 0.38 | 0.46 |
| ***Clerodendrum johnstonii*** | Leaf | Verbenaceae |  |  | 0.3 |  |  | 0.1 | 0.2 |  |  | 0.09 | 0.05 | 0.08 |
| ***Coccinia mildbraedii*** | Fruit | Cucurbitaceae |  |  |  |  |  |  |  |  |  |  | 0.1 | 0.04 |
| ***Coleus goetzenii*** | Bark | Lamiaceae |  |  |  |  |  |  |  |  | 0.04 |  | 0.14 |  |
| ***Coleus goetzenii*** | Leaf | Lamiaceae |  |  |  |  |  |  |  |  |  | 0.04 |  |  |
| ***Coleus goetzenii*** | Pith | Lamiaceae |  |  |  |  |  |  |  |  |  | 0.79 |  |  |
| ***Coleus goetzenii*** | Stem | Lamiaceae |  |  |  |  |  |  |  |  |  | 0.35 | 0.19 | 0.08 |
| ***Crassocephalum ducis-aprutii*** | Leaf | Asteraceae |  |  |  |  | 0.1 |  |  |  |  |  |  |  |
| ***Cynanchum insipidum*** | Leaf | Asclepidiaceae | 1.1 | 1.1 | 1.1 |  | 1.2 | 0.1 | 0.6 | 0.9 | 0.57 | 0.53 | 0.1 | 0.34 |
| ***Cynanchum insipidum*** | Stem | Asclepidiaceae |  |  |  |  |  |  |  |  | 0.13 |  |  |  |
| ***Cyperus cf. karisimbiensis*** | Flower | Cypeaceae |  |  |  | 0.2 |  |  | 0.2 |  | 0.04 |  |  |  |
| ***Cyperus cf. karisimbiensis*** | Leaf | Cypeaceae | 0.2 |  |  | 0.9 | 6.6 | 1.8 | 1.2 | 1.5 | 0.13 |  | 0.05 |  |
| ***Dendrosenecio erici-rosenii*** | Leaf | Asteraceae |  | 0.2 |  |  |  |  |  |  |  |  |  |  |
| ***Dendrosenecio erici-rosenii*** | Pith | Asteraceae | 3.7 | 4.2 |  | 1.8 | 0.9 | 0.2 |  | 0.7 |  |  |  |  |
| ***Dendrosenecio erici-rosenii*** | Root | Asteraceae |  |  |  |  |  |  |  | 0.5 |  |  |  |  |
| ***Dendrosenecio erici-rosenii*** | Stem | Asteraceae |  |  |  |  |  | 0.2 |  |  |  |  |  |  |
| ***Discopodium penninervium*** | Bark | Solanaceae |  |  |  |  |  |  |  |  |  |  |  | 0.08 |
| ***Discopodium penninervium*** | Dead wood | Solanaceae |  |  |  |  |  |  |  |  |  |  | 0.1 |  |
| ***Discopodium penninervium*** | Fruit | Solanaceae |  |  |  |  |  |  |  |  |  |  |  | 0.04 |
| ***Discopodium penninervium*** | Pith | Solanaceae | 0.2 |  |  | 1.4 |  | 5.7 | 0.7 | 2.1 |  | 0.04 | 0.76 | 2.07 |
| ***Discopodium penninervium*** | Stem | Solanaceae |  |  |  |  |  |  |  |  |  |  | 0.1 | 0.25 |
| ***Discopodium penninervium*** | Leaf | Solanaceae |  |  | 0.3 |  |  | 0.1 |  |  |  |  |  |  |
| ***Dombeya torrida subsp. torrida*** *^N^* | Bark | Marvaceae |  |  |  |  |  |  |  |  | 0.18 |  |  |  |
| ***Dombeya torrida subsp. torrida*** *^N^* | Flower | Marvaceae |  |  |  |  |  |  |  |  | 0.04 |  | 0.1 | 0.25 |
| ***Dombeya torrida subsp. torrida*** *^N^* | Sap | Marvaceae |  |  |  |  |  |  |  |  | 0.22 |  |  |  |
| ***Dovyalis macrocalyx (Oliv.) Warb.*** *^N^* | Fruit | Fumariaceae |  |  |  |  |  |  |  |  |  |  | 0.05 |  |
| ***Dracaena afromontana*** *^N^* | Fruit | Agavaceae |  |  |  |  |  |  |  |  | 0.04 |  |  |  |
| ***Dracaena afromontana*** *^N^* | Leaf | Agavaceae |  |  |  |  |  |  |  |  | 0.04 |  |  |  |
| ***Dracaena afromontana*** *^N^* | Root | Agavaceae |  |  |  |  |  |  |  |  |  |  |  | 0.04 |
| ***Droguetia iners*** | Cuticle | Urticaceae |  |  |  |  |  |  |  |  |  | 0.04 |  |  |
| ***Droguetia iners*** | Flower | Urticaceae |  |  |  |  |  |  |  |  | 0.04 |  |  |  |
| ***Droguetia iners*** | Leaf | Urticaceae | 2.1 | 0.9 | 2.1 | 2.5 | 1.5 | 0.5 | 1.5 | 2.1 | 3.8 | 1.31 | 1.05 | 0.46 |
| ***Droguetia iners*** | Stem | Urticaceae |  |  | 0.5 |  |  | 0.4 | 0.2 | 0.3 | 1.72 | 0.35 | 0.24 | 0.17 |
| ***Droguetia iners*** | Root | Urticaceae |  |  |  |  |  |  |  | 0.2 |  |  |  |  |
| ***Echinops hoehnelii*** | Cutic | Asteraceae |  |  |  | 0.2 |  |  |  |  |  |  |  |  |
| ***Echinops hoehnelii*** | Leaf | Asteraceae |  |  |  |  |  | 0.2 | 1.1 |  |  |  |  |  |
| ***Englerina spp*** | Bark | Loranthaceae |  |  |  |  |  |  |  |  | 0.04 | 0.04 |  |  |
| ***Englerina spp*** | Flower | Loranthaceae |  |  |  |  |  |  |  |  | 0.09 | 0.09 | 0.1 | 0.13 |
| ***Englerina spp*** | Leaf | Loranthaceae | 0.2 |  |  |  |  | 0.2 | 0.2 |  | 0.18 | 0.22 | 0.53 | 0.93 |
| ***Englerina spp*** *^N2^* | Stem | Loranthaceae |  |  |  |  |  |  |  |  | 0.09 | 0.39 | 1.96 | 2.03 |
| ***Eucalyptus species*** | Bark | Myrtaceae |  | 2.9 |  | 1.1 |  | 0.4 | 0.1 |  | 2.96 | 1.01 | 1.48 |  |
| ***Eucalyptus species*** | Dead wood | Myrtaceae |  |  |  |  |  |  |  |  | 0.93 | 0.31 | 0.43 |  |
| ***Eucalyptus species*** | Root | Myrtaceae |  |  |  |  |  |  |  |  |  | 0.04 |  |  |
| ***Eucalyptus species*** | Sap | Myrtaceae |  |  |  |  |  |  |  |  | 2.69 | 7.71 | 8.7 |  |
| ***Feces*** | All |  |  |  |  |  |  |  |  |  |  | 0.09 | 0.05 | 0.08 |
| ***Ferns*** | Cuticle |  |  |  |  |  |  |  |  |  |  |  |  | 0.04 |
| ***Ferns*** | Leaf |  |  |  |  |  |  |  |  | 0.2 |  |  |  | 0.04 |
| ***Ferns*** | Pith |  |  |  |  |  |  | 0.4 | 0.1 | 0.2 |  | 0.31 | 0.48 | 0.97 |
| ***Ferns*** | Rhizome |  |  |  |  |  |  |  |  | 0.2 |  | 0.09 | 0.14 | 0.3 |
| ***Ferns*** | Stalk |  |  |  |  |  |  |  |  |  |  | 0.48 | 0.67 | 1.14 |
| ***Ferns*** | Stalk |  |  |  |  |  |  |  | 2.1 | 0.3 |  |  |  |  |
| ***Galiniera saxiflaga*** *^N^* | Bark | Rubiaceae |  |  |  |  |  |  |  |  |  |  | 0.24 | 0.04 |
| ***Galiniera saxiflaga*** *^N^* | Fruit | Rubiaceae |  |  |  |  |  |  |  |  | 0.31 | 0.39 | 0.29 |  |
| ***Galiniera saxiflaga*** *^N^* | Pith | Rubiaceae |  |  |  |  |  |  |  |  |  | 0.09 |  | 0.08 |
| ***Galium spp*** | All | Rubiaceae | 22.6 | 23 | 30.3 | 19.7 | 18.5 | 26.2 | 20.9 | 25.3 | 3.09 | 2.06 | 2.15 | 2.03 |
| ***Galium spp*** | Cuticle | Rubiaceae |  |  |  |  |  |  |  |  |  |  | 0.05 | 0.04 |
| ***Galium spp*** | Leaf | Rubiaceae |  |  | 0.3 |  |  |  | 0.1 |  | 0.09 | 0.04 | 0.19 | 0.04 |
| ***Galium spp*** | Stem | Rubiaceae |  |  |  | 0.2 |  |  |  |  | 0.09 |  | 0.14 |  |
| ***Girardinia bulosa*** | Cuticle | Urticaceae |  |  |  |  |  |  |  |  |  |  |  | 0.04 |
| ***Girardinia bulosa*** | Dead stem | Urticaceae |  |  |  |  |  |  |  |  |  | 0.04 |  |  |
| ***Girardinia bulosa*** | Root | Urticaceae |  |  |  |  |  |  |  |  | 0.04 | 0.04 |  | 0.04 |
| ***Girardinia bulosa*** *^N^* | Stem | Urticaceae |  |  |  |  |  |  |  |  | 0.26 |  |  | 0.08 |
| ***Girardinia diversifolia*** *^N^* | Stem | Urticaceae |  |  |  |  |  |  |  |  |  |  |  | 0.04 |
| ***Girardinia diversifolia*** *^N^* | Root | Urticaceae |  |  |  |  |  |  |  |  |  | 0.04 |  |  |
| ***Hagenia abyssinica*** | Stem | Urticaceae |  |  |  |  |  |  |  |  | 0.13 |  |  |  |
| ***Hagenia abyssinica*** | Bark | Rosaceae |  |  |  |  |  |  | 0.7 |  |  |  |  |  |
| ***Helichrysum cf. foetidum*** | Leaf | Asteraceae | 0.2 |  |  |  |  |  |  |  |  |  |  |  |
| ***Helichrysum formosissimum*** | Leaf | Asteraceae | 0.3 | 0.5 | 0.3 |  | 0.2 |  |  | 0.2 |  |  |  |  |
| ***Helichrysum maraguense*** | Leaf | Asteraceae |  |  |  |  |  |  | 0.3 | 0.2 | 0.13 |  |  | 0.04 |
| ***Hylodesmum repandum*** *^N^* | Leaf | Fabaceae |  |  |  |  |  |  |  |  | 0.04 | 0.09 |  |  |
| ***Hylodesmum repandum*** *^N^* | Stem | Fabaceae |  |  |  |  |  |  |  |  |  | 0.09 |  |  |
| ***Hypericum cf. peplidifolium*** | Bark | Hypericaceae |  |  |  |  |  |  | 0.3 |  |  |  |  |  |
| ***Hypericum cf. peplidifolium*** | Root | Hypericaceae | 0.2 |  |  |  |  |  |  |  |  |  |  |  |
| ***Hypericum lanceolatum ^1^*** | Bark | Hypericaceae |  |  |  |  |  |  | 0.2 |  |  |  |  |  |
| ***Ipomoea involucrata*** *^N^* | Cuticle | Convolvulaceae |  |  |  |  |  |  |  |  |  | 0.04 | 0.05 |  |
| ***Ipomoea involucrata*** *^N^* | Leaf | Convolvulaceae |  |  |  |  |  |  |  |  |  |  | 0.1 | 0.3 |
| ***Juniperus procera*** | Sap | Cupressaceae |  |  |  |  |  |  |  |  | 0.18 |  |  |  |
| ***Juniperus procera*** *^N^* | Bark | Cupressaceae |  |  |  |  |  |  |  |  | 0.84 |  |  |  |
| ***Lactuca spp*** | Flower | Asteraceae |  |  |  |  |  |  |  |  | 0.13 |  | 0.1 |  |
| ***Lactuca spp*** | Leaf | Asteraceae |  |  |  |  |  |  |  |  | 0.31 |  | 0.19 | 0.25 |
| ***Lactuca spp*** | Stem | Asteraceae |  |  |  |  |  |  |  |  | 0.26 |  | 0.1 | 0.04 |
| ***Lactuca spp*** | Cutic | Asteraceae |  |  |  |  |  |  | 0.1 |  |  |  |  |  |
| ***Lactuca spp*** | Flower | Asteraceae |  |  |  |  |  | 0.1 |  |  |  |  |  |  |
| ***Lactuca spp*** | Leaf | Asteraceae |  |  |  |  |  | 0.1 | 0.5 | 0.2 |  |  |  |  |
| ***Lactuca spp*** | Stem | Asteraceae |  |  |  |  |  | 0.1 | 0.1 |  |  |  |  |  |
| ***Lactuca spp*** *^N3^* | Cutic | Asteraceae |  |  |  |  |  |  |  |  | 0.04 |  |  |  |
| ***Laportea alatipes*** | Leaf | Urticaceae | 3.8 | 3.3 | 1.8 | 3 | 1.2 | 6.2 | 4 | 3.2 | 0.26 |  | 0.05 | 0.13 |
| ***Laportea alatipes*** | Root | Urticaceae |  |  |  | 0.2 |  | 0.2 | 1.1 | 1.2 | 0.71 | 0.18 | 0.48 | 0.42 |
| ***Laportea alatipes*** | Stem | Urticaceae | 2.4 |  |  | 0.5 |  | 0.7 | 1.6 | 0.9 | 0.22 | 0.04 | 0.14 | 0.04 |
| ***Lobelia giberroa*** | Root | Lobeliaceae |  |  |  |  |  |  |  | 0.2 |  |  |  |  |
| ***Lobelia giberroa*** | Stem | Lobeliaceae |  |  |  |  | 2.2 |  | 0.2 |  |  |  |  |  |
| ***Lobelia stuhlmanii*** | Bark | Lobeliaceae | 3.4 | 0.5 |  |  |  | 0.1 |  |  |  |  |  |  |
| ***Lobelia stuhlmanii*** | Stem | Lobeliaceae |  | 0.5 |  | 1.3 | 0.1 | 0.1 | 0.2 | 0.3 |  |  |  |  |
| ***Lobelia wollastonii*** | Stem | Lobeliaceae |  | 0.2 |  |  |  |  |  |  |  |  |  |  |
| ***Maesa latifolia*** *^N^* | Bark | Urticaceae |  |  |  |  |  |  |  |  |  |  | 0.33 | 0.04 |
| ***Maesa latifolia*** *^N^* | Dry bark | Primulaceae |  |  |  |  |  |  |  |  |  | 0.04 |  |  |
| ***Maesa latifolia*** *^N^* | Dead wood | Primulaceae |  |  |  |  |  |  |  |  |  | 0.04 |  | 0.04 |
| ***Maesa latifolia*** *^N^* | Leaf | Primulaceae |  |  |  |  |  |  |  |  |  |  |  | 0.08 |
| ***Maesa latifolia*** *^N^* | Stem | Primulaceae |  |  |  |  |  |  |  |  |  |  |  | 0.21 |
| ***Mikania chenopodiifolia Willd.*** | Bark | Asteraceae |  |  |  |  |  |  |  |  |  | 0.09 | 0.43 | 0.46 |
| ***Mikania chenopodiifolia Willd.*** | Flower | Asteraceae |  |  |  |  |  |  |  |  |  |  |  | 0.04 |
| ***Mikania chenopodiifolia Willd.*** | Leaf | Asteraceae |  |  |  |  |  | 0.4 |  |  |  | 0.13 | 0.05 | 0.17 |
| ***Mikaniopsis sp.*** *^N^* | Cuticle | Acanthaceae |  |  |  |  |  |  |  |  | 0.04 |  |  |  |
| ***Mimulopsis arborescens*** *^N^* | Pith | Primulaceae |  |  |  |  |  |  |  |  |  |  |  | 0.04 |
| ***Mimulopsis solmsii*** | Bark | Acanthaceae |  |  |  |  |  |  |  |  |  | 0.04 |  | 0.08 |
| ***Mimulopsis solmsii*** | Flower | Acanthaceae |  |  |  |  |  |  |  |  |  | 0.26 |  | 0.38 |
| ***Mimulopsis solmsii*** | Root | Acanthaceae |  |  |  |  |  |  |  |  |  | 0.04 |  |  |
| ***Mimulopsis solmsii*** | Stem | Acanthaceae |  |  |  |  |  |  |  |  | 0.18 |  |  | 0.04 |
| ***Musa acuminata*** *^N^* | Leaf sheath | Musaceae |  |  |  |  |  |  |  |  |  |  | 0.76 |  |
| ***Mushroom*** | All |  |  |  |  |  |  | 0.6 | 0.2 | 0.3 |  |  |  |  |
| ***Mushroom 1*** | All |  |  |  |  |  |  |  |  |  | 0.04 | 0.26 | 0.1 | 0.34 |
| ***Mushroom 2*** | All |  |  |  |  |  |  |  |  |  | 0.04 | 0.04 | 0.05 | 0.25 |
| ***Myrsine melanophloeos*** | Bark | Myrsinaceae |  |  |  |  |  |  |  |  | 0.04 | 0.04 |  | 0.04 |
| ***Myrsine melanophloeos*** | Deadwood | Myrsinaceae |  |  |  |  |  |  |  |  |  | 0.18 | 0.05 |  |
| ***Myrsine melanophloeos*** | Fruit | Myrsinaceae |  |  |  |  |  |  |  |  |  | 0.09 |  |  |
| ***Myrsine melanophloeos*** | Leaf | Myrsinaceae |  |  |  |  |  |  |  |  |  | 0.13 |  | 0.04 |
| ***Myrsine melanophloeos*** | Stem | Myrsinaceae |  |  |  |  |  |  |  |  |  | 0.09 |  | 0.04 |
| ***Neoboutonia macrocalyx*** *^N^* | Bark | Urticaceae |  |  |  |  |  |  |  |  | 0.04 | 0.04 | 0.05 | 0.13 |
| ***Neoboutonia macrocalyx*** *^N^* | Dead wood | Urticaceae |  |  |  |  |  |  |  |  |  |  | 0.1 | 0.21 |
| ***Neoboutonia macrocalyx*** *^N^* | Pith | Urticaceae |  |  |  |  |  |  |  |  |  |  |  | 0.08 |
| ***Neoboutonia macrocalyx*** *^N^* | Sap | Urticaceae |  |  |  |  |  |  |  |  | 0.04 |  | 0.19 | 0.21 |
| ***Oldeania alpina*** | Dead shoot | Poaceae |  |  |  |  |  |  |  |  | 0.13 | 0.22 | 0.43 | 0.17 |
| ***Oldeania alpina*** | Dead stem | Poaceae |  |  |  |  |  |  |  |  |  |  |  | 0.13 |
| ***Oldeania alpina*** | Flower | Poaceae |  |  |  |  |  |  |  |  | 0.04 |  |  |  |
| ***Oldeania alpina*** | Leaf | Poaceae | 0.2 | 0.2 |  | 0.9 | 0.9 | 1.9 | 1.4 |  | 12.84 | 7.71 | 6.02 | 8.19 |
| ***Oldeania alpina*** | Shoot | Poaceae | 15 | 14.6 | 17.1 | 9.9 | 18.8 | 3 | 4 |  | 21.71 | 17.69 | 19.21 | 16.64 |
| ***Oldeania alpina*** | Stem | Poaceae | 0.2 | 0.4 |  |  |  |  |  |  | 0.66 | 0.22 | 0.62 | 0.25 |
| ***Pavetta rwandensis Bridson*** *^N^* | Fruit | Rubiaceae |  |  |  |  |  |  |  |  |  | 0.22 |  | 0.68 |
| ***Pavetta rwandensis Bridson*** *^N^* | Pith | Rubiaceae |  |  |  |  |  |  |  |  |  |  |  | 0.04 |
| ***Pilea bambuseti*** *^N^* | Stem | Urticaceae |  |  |  |  |  |  |  |  |  |  |  | 0.08 |
| ***Pilea rivularis*** | Cuticle | Urticaceae |  |  |  |  |  |  |  |  | 0.13 |  | 0.14 |  |
| ***Pilea rivularis*** | Leaf | Urticaceae |  |  |  |  | 1.9 | 0.1 | 0.1 |  | 0.09 | 0.13 | 0.05 | 0.04 |
| ***Pilea rivularis*** | Stem | Urticaceae |  |  |  |  |  |  |  |  | 0.26 | 0.22 | 0.38 | 0.38 |
| ***Pilea rivularis*** | Pith | Urticaceae |  |  |  |  |  |  |  |  | 0.04 |  | 0.05 |  |
| ***Pilea rivularis*** | Stem | Urticaceae |  |  |  |  |  |  |  |  |  |  | 0.1 |  |
| ***Plagiochila squamulosa*** | All | Selaginellaceae |  |  |  |  |  |  |  |  |  |  | 0.05 | 0.21 |
| ***Plectranthus sp.*** *^N4^* | Stem | Lamiaceae |  |  |  |  |  |  |  |  |  | 0.04 |  |  |
| ***Pleopeltis macrocarpa*** | All | Polypodiaceae |  |  |  |  |  | 0.4 |  | 0.5 | 0.04 | 0.04 | 1.86 | 4.22 |
| ***Pleopeltis macrocarpa*** | Leaf | Polypodiaceae |  | 0.2 |  |  |  |  | 0.1 |  |  |  |  |  |
| ***Prunus africana^2^*** | Bark | Rosaceae |  |  |  |  |  |  |  |  |  | 0.04 |  |  |
| ***Prunus africana^2^*** | Deadwood | Rosaceae |  |  |  |  |  |  |  |  |  | 0.09 | 0.14 | 0.13 |
| ***Prunus africana^2^*** | Leaf | Rosaceae |  |  |  |  |  |  |  |  |  |  |  | 0.08 |
| ***Prunus africana^2^*** | Stem | Rosaceae |  |  |  |  |  |  |  |  |  |  |  | 0.08 |
| ***Psychotria mahonii*** | Dry bark | Rubiaceae |  |  |  |  |  |  |  |  |  | 0.04 |  |  |
| ***Psychotria mahonii*** | Stem | Rubiaceae |  |  |  |  |  |  |  |  |  | 0.13 |  |  |
| ***Psychotria mahonii*** *^N^* | Fruit | Rubiaceae |  |  |  |  |  |  |  |  |  | 0.04 | 0.1 | 0.42 |
| ***Psychotria mahonii*** *^N^* | Leaf | Rubiaceae |  |  |  |  |  |  |  |  | 0.09 |  |  |  |
| ***Psychotria mahonii*** *^N^* | Pith | Rubiaceae |  |  |  |  |  |  |  |  |  |  |  | 0.04 |
| ***Psychotria mahonii*** *^N^* | Bark | Rubiaceae |  |  |  |  |  |  |  |  |  | 0.04 |  | 0.04 |
| ***Rubia cordifolia*** *^N^* | All | Rubiaceae |  |  |  |  |  |  |  |  |  |  | 0.05 |  |
| ***Rubus species*** | Fruit | Rubiaceae | 0.2 | 0.7 |  | 0.5 | 0.2 |  | 0.7 | 0.9 | 0.04 | 0.13 | 0.05 | 0.13 |
| ***Rubus species*** | Leaf | Rubiaceae | 1.4 | 2.7 | 6.1 | 2 | 5.4 | 3.5 | 4 | 1.9 | 0.57 | 0.83 | 1.29 | 0.51 |
| ***Rubus species*** | Root | Rubiaceae |  |  |  |  |  |  |  |  |  |  |  | 0.04 |
| ***Rubus species*** | Stem | Rubiaceae |  |  |  |  |  |  |  |  |  | 0.04 | 0.05 | 0.04 |
| ***Rumex abyssinicus*** | Leaf | Polygonaceae | 0.2 |  |  |  | 0.2 |  |  |  |  |  |  |  |
| ***Rumex abyssinicus*** | Stem | Polygonaceae | 0.3 | 0.4 |  |  | 0.2 |  |  |  |  |  |  |  |
| ***Rumex nepalensis var. nepalensis*** | Stem | Polygonaceae |  |  |  | 0.4 |  |  |  |  |  |  |  |  |
| ***Rumex ruwenzoriensis*** | Stem | Polygonaceae |  |  |  |  | 0.1 |  |  |  |  |  |  |  |
| ***Rumex usambarensis*** | Leaf | Polygonaceae |  |  |  |  |  |  |  |  |  |  | 0.05 | 0.04 |
| ***Scepocarpus hypselodendron*** | Bark | Urticaceae |  |  |  |  |  |  |  |  | 15.09 | 10.29 | 8.55 | 10.3 |
| ***Scepocarpus hypselodendron*** | Fruit | Urticaceae |  |  |  |  |  |  |  |  |  | 0.09 | 0.29 |  |
| ***Scepocarpus hypselodendron*** | Leaf | Urticaceae |  |  |  | 0.2 |  |  |  | 0.2 | 6.18 | 9.15 | 5.16 | 5.74 |
| ***Scepocarpus hypselodendron*** | Pith | Urticaceae |  |  |  |  |  |  |  |  | 0.04 | 0.13 |  |  |
| ***Scepocarpus hypselodendron*** | Stem | Urticaceae |  |  |  |  |  |  |  |  |  | 0.18 | 0.14 | 0.34 |
| ***Secamone africana*** | Cuticle | Apocynaceae |  |  |  |  |  |  |  |  |  | 0.04 | 0.05 | 0.08 |
| ***Secamone africana*** *^N^* | Leaf | Apocynaceae |  |  |  |  |  |  |  |  | 0.84 | 5.74 | 5.78 | 9.12 |
| ***Secamone africana*** *^N^* | Stem | Apocynaceae |  |  |  |  |  |  |  |  | 0.13 | 0.48 | 0.38 | 0.25 |
| ***Senecio maranguensis*** | Bark | Asteraceae |  |  |  |  |  |  |  |  |  |  | 0.14 |  |
| ***Senecio maranguensis*** | Root | Asteraceae |  |  |  |  |  |  |  |  | 0.04 |  |  |  |
| ***Senecio maranguensis*** | Stem | Asteraceae |  |  |  |  |  |  |  |  | 0.13 |  |  | 0.08 |
| ***Senecio maranguensis*** *^N^* | Leaf | Asteraceae |  |  |  |  |  |  |  |  |  |  |  | 0.08 |
| ***Senecio maranguensis*** *^N^* | Pith | Asteraceae |  |  |  |  |  |  |  |  | 0.31 | 0.04 | 0.48 | 0.42 |
| ***Senecio mariettae*** | Pith | Asteraceae |  |  |  |  |  |  |  |  | 0.04 |  |  | 0.13 |
| ***Senecio mariettae*** | Root | Asteraceae |  |  |  |  |  |  |  |  | 0.13 |  |  |  |
| ***Senecio mariettae*** | Stem | Asteraceae |  |  |  |  |  |  |  |  | 0.04 |  |  |  |
| ***Solanecio manii*** | Dry stem | Asteraceae |  |  |  |  |  |  |  |  |  | 0.09 | 0.05 | 0.63 |
| ***Solanecio manii*** | Flower | Asteraceae |  |  |  |  |  |  |  |  |  |  |  | 0.04 |
| ***Solanecio manii*** *^N^* | Pith | Asteraceae |  |  |  |  |  |  |  |  |  | 0.13 | 0.1 | 1.01 |
| ***Solanum betaceum*** *^N^* | Fruit | Solanaceae |  |  |  |  |  |  |  |  |  |  | 0.81 |  |
| **Thistle** *^N1^* | Flower | Asteraceae |  | 0.4 |  | 0.4 | 1.7 |  | 0.8 | 0.2 | 0.44 | 0.53 | 0.53 | 0.68 |
| **Thistle** *^N1^* | Leaf | Asteraceae | 17.1 | 19.3 | 23.4 | 34.8 | 23.9 | 15.2 | 22.8 | 17.3 | 6.31 | 7.31 | 2.72 | 2.91 |
| **Thistle** *^N1^* | Root | Asteraceae | 2 | 0.5 | 6.6 | 4.1 | 4.5 | 8.8 | 14.5 | 4.3 | 3.05 | 6 | 2.77 | 1.86 |
| **Thistle** *^N1^* | Stem | Asteraceae | 2 | 6.9 | 1.8 | 2.7 | 3.1 | 3.9 | 2 | 2.6 | 2.21 | 1.36 | 1.24 | 1.27 |
| ***Unidentified 10*** | Leaf |  |  |  |  |  |  |  |  |  |  |  |  | 0.04 |
| ***Unidentified 10*** | Rhizome |  |  |  |  |  |  |  |  |  |  |  |  | 0.04 |
| ***Unidentified 4*** | Leaf |  |  |  |  |  |  |  |  |  |  |  | 0.14 |  |
| ***Urtica massaica*** | Leaf | Urticaceae |  |  | 0.3 |  | 2.8 | 0.1 | 1.2 |  | 0.13 | 0.04 |  | 0.04 |
| ***Urtica massaica*** | Root | Urticaceae |  |  |  |  |  |  |  |  | 0.04 |  |  |  |
| ***Urtica massaica*** | Stem | Urticaceae |  |  |  |  |  | 0.5 | 0.1 | 0.2 |  |  |  | 0.04 |
| ***Vasconcellea pubescens*** *^N^* | Fruit | Caricaceae |  |  |  |  |  |  |  |  |  |  | 0.67 |  |
| ***Xymalos monospora*** | Bark | Monimiaceae |  |  |  |  |  |  |  |  |  |  | 0.05 |  |
| ***Xymalos monospora*** | Deadwood | Monimiaceae |  |  |  |  |  |  |  |  |  |  | 0.05 |  |
| **N food type-items** | |  | **24** | **23** | **18** | **25** | **26** | **39** | **43** | **39** | **86** | **94** | **105** | **123** |
| **N food types** | |  | **17** | **15** | **12** | **16** | **19** | **25** | **27** | **24** | **40** | **42** | **52** | **54** |
| **N feeding observations** | | | **655** | **548** | **380** | **558** | **895** | **828** | **996** | **585** | **2266** | **2284** | **2093** | **2368** |

***^1^ (syn. revolutum), ^2^(syn. Pygeum africanum),*** *^N^ newly recorded food type-item, ^N1^ contains items of new species: Cirsium dissectum, ^N2^ contains item of new but unidentified Engerlina species, ^N3^ contains new but unidentified species of Lactuca, ^N4^ contains item of a new but unidentified Plectranthus.*

Supplementary Table 4. Newly identified mountain gorilla food type-items that were only recorded through opportunistic sampling in this study.

| **Food item** | **Part** | **Family** | **SAB** | **MUH** | **KSA** | **KWI** |
| --- | --- | --- | --- | --- | --- | --- |
| *Conyza stricta Willd.* | Leaf | Asteraceae | |  | X |  |
| *Cynoglossum amplifolius* | Leaf | Boraginaceae | X |  |  |  |
| *Cyperus brevifolius* | Leaf | Cyperaceae | X |  |  |  |
| *Cyperus brevifolius* | Flower | Cyperaceae | X |  |  |  |
| *Cyperus brevifolius* | Root | Cyperaceae | X |  |  |  |
| *Euphorbia schimperiana var. schimperiana* | Leaf | Euphorbiaceae | |  |  |  |
| *Euphorbia schimperiana var. schimperiana* | Stem | Euphorbiaceae | |  |  |  |
| *Hoffmannanthus abbotianus O.Hoffm* | Stem | Asteraceae | |  | X |  |
| *Hoffmannanthus abbotianus O.Hoffm* | Pith | Asteraceae | |  | X |  |
| *Kniphofia bequaertii* | Stem | Xanthorrhoeaceae | |  | X |  |
| *Lactuca cf. glandulifera* | Leaf | Asteraceae | |  |  | X |
| *Maytenus acuminata* | Stem | Celastraceae | | X |  |  |
| *Maytenus acuminata* | Root | Celastraceae | | X |  |  |
| Mushroom 3 | All |  |  |  |  | X |
| *Passiflora ligularis Juss.* | Fruit | Passifloraceae | |  |  | X |
| *Piper capense* | Pith | Piperaceae | |  | X | X |
| *Piper capense* | Stem | Piperaceae | |  | X | X |
| *Rhamnus prinoides* | Fruit | Rhamnaceae | | X |  |  |
| *Rhamnus prinoides* | Leaf | Rhamnaceae | | X |  |  |
| *Solanum terminale* | Fruit | Solanaceae | |  |  |  |
| *Sonchus oleraceus* | Leaf | Asteraceae | |  | X |  |
| *Sonchus oleraceus* | Stem | Asteraceae | |  | X |  |
| Unidentified 1 | Leaf |  |  |  |  | X |
| Unidentified 6 | Fruit |  |  | X |  |  |
| Unidentified 7 | Leaf |  |  |  |  |  |
| Unidentified 7 | Flower |  |  |  |  |  |
| Unidentified 8 | Stem |  | X |  |  |  |
| Unidentified 9 | All |  | X |  |  |  |
| **Insects** |  |  |  |  |  |  |
| Termites | All |  |  |  |  | X |
| Unidentified insects | All |  |  | X |  |  |

Supplementary Table 5. Updated list of foods consumed by mountain gorilla in the Virunga massif and Bwindi Impenetrable National Park.

(provided in a separate Excel file)

Supplementary Table 6. Food type-item combinations making up ~80% of the diet and Hill-Shannon diet diversity ‘outside the park’ presented by study group (southwest groups in grey, northeast groups in white) sorted from left to right by their location in the Volcanoes National Park from the furthest southwest (DUS=Dushishoze) to the furthest east (KWI=Kwitonda).

| **Food item** | **Part** | **Plant family** | **DUS** | **PAB** | **MSK** | **KRB** | **TOB** | **TIT** | **SEG** | **NTA** | **SAB** | **MUH** | **KSA** | **KWI** |
| --- | --- | --- | --- | --- | --- | --- | --- | --- | --- | --- | --- | --- | --- | --- |
| *Acacia melanoxylon* | Bark | Fabaceae |  |  |  |  |  |  |  |  |  |  |  | 22.56 |
| *Acacia melanoxylon* | Dry bark | Fabaceae |  |  |  |  |  |  |  |  |  |  |  | 5.26 |
| *Acacia melanoxylon* | Dead wood | Fabaceae |  |  |  |  |  |  |  |  |  |  |  | 7.52 |
| *Acacia melanoxylon* | Root | Fabaceae |  |  |  |  |  |  |  |  |  |  |  | 4.51 |
| *Acacia melanoxylon* | Stem | Fabaceae |  |  |  |  |  |  |  |  |  |  |  | 18.05 |
| *Acacia mearnsii* | Leaf | Fabaceae |  |  |  |  |  |  |  |  |  |  | 10.07 |  |
| *Acacia mearnsii* | Stem | Fabaceae |  |  |  |  |  |  |  |  |  |  | 6.13 |  |
| Thistles | Leaf | Asteraceae |  |  |  |  |  | 20 |  |  | 3.59 |  |  |  |
| *Englerina species* | Leaf | Loranthaceae |  |  |  |  |  |  |  |  |  |  | 2.19 | 9.77 |
| *Englerina species* | Stem | Loranthaceae |  |  |  |  |  |  |  |  |  |  | 5.25 | 14.29 |
| *Eucalyptus species* | Bark | Myrtaceae |  | 94.12 |  | 85.71 |  | 60 |  |  | 34.36 | 9.54 | 6.78 |  |
| *Eucalyptus species* | Dead wood | Myrtaceae |  |  |  |  |  |  |  |  | 10.77 |  |  |  |
| *Eucalyptus species* | Sap | Myrtaceae |  |  |  |  |  |  |  |  | 31.28 | 73.03 | 39.82 |  |
| *Musa acuminata* | Leaf-sheath | Musaceae |  |  |  |  |  |  |  |  |  |  | 3.50 |  |
| *Rubus spp.* | Leaf | Rubiaceae |  |  |  |  |  |  |  |  |  |  | 2.63 |  |
| *Solanum betaceum* | Fruit | Solanaceae |  |  |  |  |  |  |  |  |  |  | 3.28 |  |
| *Vaconcelea pubescens* | Fruit | Caricaceae |  |  |  |  |  |  |  |  |  |  | 3.06 |  |
| **N feeding observations** |  |  | **0** | **17** | **0** | **7** | **0** | **5** | **0** | **0** | **195** | **241** | **457** | **133** |
| **% covered by key foods** |  |  |  | **94.12** |  | **85.71** |  | **80.00** |  |  | **80.00** | **82.57** | **82.71** | **81.95** |
| **Diet diversity** |  |  |  | **0.050** |  | **0.168** |  | **0.903** |  |  | **3.861** | **1.272** | **6.150** | **5.856** |

Supplementary Table 7. Food type-item combinations making up ~80% of the diet and Hill-Shannon diet diversity in the ‘mixed forest zone’ presented by study group (southwest groups in grey, northeast groups in white) sorted from left to right by their location in the Volcanoes National Park from the furthest southwest (DUS=Dushishoze) to the furthest east (KWI=Kwitonda).

| **Food item** | **Part** | **Plant family** | **DUS** | **PAB** | **MSK** | **KRB** | **TOB** | **TIT** | **SEG** | **NTA** | **SAB** | **MUH** | **KSA** | **KWI** |
| --- | --- | --- | --- | --- | --- | --- | --- | --- | --- | --- | --- | --- | --- | --- |
| *Basella alba* | Leaf | Basellaceae |  |  |  |  |  |  |  |  |  |  | 3.49 | 3.87 |
| Thistles | Leaf | Asteraceae |  |  | 11.11 |  |  |  |  |  |  |  | 1.55 | 1.94 |
| *Clematis simensis* | Pith | Ranunculceae |  |  |  |  |  |  |  |  |  |  | 17.44 | 15.48 |
| *Discopodium penninervium* | Pith | Solanaceae |  |  |  |  |  |  |  |  |  |  | 3.10 | 3.74 |
| *Englerina* species | Stem | Loranthaceae |  |  |  |  |  |  |  |  |  |  | 4.65 | 1.42 |
| Ferns | Pith | - |  |  |  |  |  |  |  |  |  |  |  | 1.55 |
| Ferns | Stalk | - |  |  |  |  |  |  |  |  |  |  |  | 2.32 |
| *Galium spp* | All | Rubiaceae |  |  | 77.78 |  |  |  |  |  |  |  | 4.65 | 2.71 |
| *Pleopertis macrocarpa* | All | Polypodiaceae |  |  |  |  |  |  |  |  |  |  | 8.53 | 10.19 |
| *Secamone africana* | Leaf | Apocynaceae |  |  |  |  |  |  |  |  |  |  | 17.44 | 12.52 |
| *Solanecio manii* | Pith | Solanaceae |  |  |  |  |  |  |  |  |  |  |  | 2.45 |
| *Scepocarpus hypselodendron* | Bark | Urticaceae |  |  |  |  |  |  |  |  |  |  | 15.12 | 14.71 |
| *Scepocarpus hypselodendron* | Leaf | Urticaceae |  |  |  |  |  |  |  |  |  |  | 4.65 | 7.74 |
| **N feeding observations** |  |  | **0** | **0** | **9** | **0** | **0** | **0** | **0** | **0** | **0** | **0** | **258** | **775** |
| **% covered by key foods** |  |  |  |  | **88.89** |  |  |  |  |  |  |  | **80.62** | **80.65** |
| **Diet diversity** |  |  |  |  | **0.468** |  |  |  |  |  |  |  | **8.277** | **5.856** |

Supplementary Table 8. Food type-item combinations making up ~80% of the diet and Hill-Shannon diet diversity in the ‘bamboo/mixed bamboo zone’ presented by study group (southwest groups in grey, northeast groups in white) sorted from left to right by their location in the Volcanoes National Park from the furthest southwest (DUS=Dushishoze) to the furthest east (KWI=Kwitonda).

| **Food item** | **Part** | **Plant family** | **DUS** | **PAB** | **MSK** | **KRB** | **TOB** | **TIT** | **SEG** | **NTA** | **SAB** | **MUH** | **KSA** | **KWI** |
| --- | --- | --- | --- | --- | --- | --- | --- | --- | --- | --- | --- | --- | --- | --- |
| *Oldeania alpina* | Leaf | Poaceae |  |  |  |  |  | 13.01 | 10.22 |  | 14.44 | 8.62 | 8.14 | 11.72 |
| *Oldeania alpina* | Shoot | Poaceae | 71.53 | 55.56 | 60.19 | 37.16 | 46.54 | 20.33 | 29.20 |  | 24.37 | 19.81 | 29.69 | 26.69 |
| *Basella alba* | Leaf | Basellaceae |  |  |  |  |  |  |  |  | 5.71 | 9.12 | 6.25 | 2.82 |
| Thistles | Leaf | Asteraceae |  |  |  | 12.84 | 14.68 | 8.94 | 16.79 |  | 6.35 | 8.13 | 3.77 | 3.46 |
| Thistles | Root | Asteraceae |  |  |  |  |  | 7.32 | 8.03 |  | 2.98 | 6.75 | 3.47 | 2.75 |
| Thistles | Stem | Asteraceae |  |  |  |  |  |  |  |  |  |  |  | 1.20 |
| *Clematis simensis* | Pith | Ranunculaceae |  |  |  |  |  |  |  |  |  |  | 4.07 | 4.45 |
| *Discopodium penninervium* | Pith | Solanaceae |  |  |  |  |  |  |  |  |  |  |  | 1.27 |
| *Droguetia iners* | Leaf | Urticaceae |  |  |  |  |  |  |  |  | 4.12 |  |  |  |
| *Galium spp* | All | Rubiaceae |  | 23.61 | 24.07 | 30.41 | 15.79 | 23.58 | 16.06 |  |  |  | 1.96 | 1.91 |
| *Laportea alatipes* | Leaf | Urticaceae | 9.49 | 9.03 |  |  |  | 12.20 |  |  |  |  |  |  |
| *Pleopertis macrocarpa* | All | Polypodiaceae |  |  |  |  |  |  |  |  |  |  |  | 1.48 |
| *Secamone africana* | Leaf | Apocynaceae |  |  |  |  |  |  |  |  |  | 6.46 | 5.73 | 8.40 |
| *Scepocarpus hypselodendron* | Bark | Urticaceae |  |  |  |  |  |  |  |  | 16.87 | 11.58 | 10.47 | 9.11 |
| *Scepocarpus hypselodendron* | Leaf | Urticaceae |  |  |  |  |  |  |  |  | 6.95 | 10.25 | 7.08 | 5.30 |
| *Urtica massaica* | Leaf | Urticaceae |  |  |  |  | 6.93 |  |  |  |  |  |  |  |
| **N feeding observations** |  |  | **137** | **144** | **108** | **148** | **361** | **123** | **137** | **0** | **2015** | **2029** | **1327** | **1416** |
| **% covered by key foods** |  |  | **81.02** | **88.19** | **84.26** | **80.41** | **83.93** | **85.37** | **80.29** |  | **81.79** | **80.73** | **80.63** | **80.58** |
| **Diet diversity** |  |  | **1.177** | **1.844** | **1.479** | **2.608** | **2.897** | **4.494** | **4.414** |  | **6.662** | **8.003** | **7.779** | **8.940** |

Supplementary Table 9. Food type-item combinations making up ~80% of the diet and Hill-Shannon diet diversity in the ‘herbaceous zone’ presented by study group (southwest groups in grey, northeast groups in white) sorted from left to right by their location in the Volcanoes National Park from the furthest southwest (DUS=Dushishoze) to the furthest east (KWI=Kwitonda).

| **Food item** | **Part** | **Plant family** | **DUS** | **PAB** | **MSK** | **KRB** | **TOB** | **TIT** | **SEG** | **NTA** | **SAB** | **MUH** | **KSA** | **KWI** |
| --- | --- | --- | --- | --- | --- | --- | --- | --- | --- | --- | --- | --- | --- | --- |
| *Basella alba* | Flower | Basellaceae |  |  |  |  |  |  |  |  | 5.26 |  |  |  |
| *Basella alba* | Leaf | Basellaceae |  |  |  |  |  |  |  |  | 5.26 | 20.00 | 31.03 |  |
| *Basella alba* | Stem | Basellaceae |  |  |  |  |  |  |  |  |  |  | 3.45 |  |
| Thistles | Leaf | Asteraceae |  |  |  |  |  |  | 57.14 |  | 26.32 |  |  |  |
| *Discopodium penninervium* | Pith | Solanaceae |  |  |  |  |  |  |  | 12.90 |  |  |  | 66.67 |
| *Droguetia iners* | Leaf | Urticaceae |  |  |  |  |  |  |  |  | 15.79 | 13.33 |  |  |
| *Ferns* | Pith | - |  |  |  |  |  |  |  |  |  |  | 6.90 |  |
| *Galium spp* | All | Rubiaceae |  |  |  |  |  |  | 28.57 |  | 10.53 | 6.67 | 24.14 |  |
| *Afroligusticum linderi* | Stem | Apiaceae |  |  |  |  |  |  |  | 74.19 | 21.05 |  | 17.24 |  |
| *Coleus goetzenii* | Pith | Lamiaceae |  |  |  |  |  |  |  |  |  | 40.00 |  |  |
| *Senecio maranguensis* | Pith | Asteraceae |  |  |  |  |  |  |  |  |  |  |  | 33.33 |
| **N feeding observations** |  |  | **0** | **0** | **0** | **0** | **0** | **0** | **7** | **31** | **19** | **15** | **29** | **3** |
| **% covered by key foods** |  |  |  |  |  |  |  |  | **85.71** | **87.10** | **84.21** | **80.00** | **82.76** | **100.00** |
| **Diet diversity** |  |  |  |  |  |  |  |  | **0.914** | **0.676** | **3.932** | **2.819** | **3.572** | **0.406** |

Supplementary Table 10. Food type-item combinations making up ~80% of the diet and Hill-Shannon diet diversity in the ‘*Hagenia-Hypericum* zone’ presented by study group (southwest groups in grey, northeast groups in white) sorted from left to right by their location in the Volcanoes National Park from the furthest southwest (DUS=Dushishoze) to the furthest east (KWI=Kwitonda).

| **Food item** | **Part** | **Plant family** | **DUS** | **PAB** | **MSK** | **KRB** | **TOB** | **TIT** | **SEG** | **NTA** | **SAB** | **MUH** | **KSA** | **KWI** |
| --- | --- | --- | --- | --- | --- | --- | --- | --- | --- | --- | --- | --- | --- | --- |
| Thistle | Leaf | Asteraceae | 23.19 | 23.20 | 28.98 | 46.44 | 28.4 | 16.52 | 23.49 | 15.2 |  |  |  |  |
| Thistle | Root | Asteraceae |  |  | 10.80 | 4.84 | 11 | 9.21 | 15.66 | 4.02 | 60.00 |  |  |  |
| *Cyperus sp.* | Leaf | Cyperaceae |  |  |  |  | 16.5 |  |  |  | 30.00 |  |  |  |
| *Discopodium penninervium* | Pith | Solanaceae |  |  |  |  |  | 6.73 |  |  |  |  |  |  |
| Ferns | Pith |  |  |  |  |  |  |  | 2.49 |  |  |  |  |  |
| *Galium spp* | All | Rubiaceae | 19.71 | 25.20 | 34.09 | 16.24 | 19.2 | 27.19 | 21.83 | 28.3 |  |  |  |  |
| *Laportea alatipes* | Leaf | Urticaceae |  |  |  |  |  | 5.26 | 4.63 | 3.17 |  |  |  |  |
| *Afroligusticum linderi* | Stem | Apiaceae | 40.58 | 34.80 | 16.48 | 16.81 | 8.84 | 18.86 | 10.91 | 31.7 |  |  |  |  |
| *Rubus spp* | Leaf | Rubiaceae |  |  |  |  |  |  | 3.08 |  |  |  |  |  |
| **N feeding observations** |  |  | **345** | **250** | **176** | **351** | **328** | **684** | **843** | **473** | **10** | **0** | **0** | **0** |
| **% covered by key foods** |  |  | **83.48** | **83.20** | **90.34** | **84.33** | **83.84** | **83.77** | **82.09** | **82.45** | **90.00** |  |  |  |
| **Diet diversity** |  |  | **2.723** | **2.522** | **2.816** | **2.969** | **4.032** | **5.180** | **5.683** | **4.194** | **0.806** |  |  |  |

Supplementary Table 11. Food type-item combinations making up ~80% of the diet and Hill-Shannon diet diversity in the ‘alpine/subalpine zone’ presented by study group (southwest groups in grey, northeast groups in white) sorted from left to right by their location in the Volcanoes National Park from the furthest southwest (DUS=Dushishoze) to the furthest east (KWI=Kwitonda).

| **Food item** | **Part** | **Plant family** | **DUS** | **PAB** | **MSK** | **KRB** | **TOB** | **TIT** | **SEG** | **NTA** | **SAB** | **MUH** | **KSA** | **KWI** |
| --- | --- | --- | --- | --- | --- | --- | --- | --- | --- | --- | --- | --- | --- | --- |
| Thistles | Leaf | Asteraceae | 17.75 | 33.58 | 41.38 | 23.08 | 33.01 | 10.00 | 14.29 | 35.80 |  |  |  |  |
| Thistles | Root | Asteraceae |  |  |  | 7.69 |  |  |  | 7.41 |  |  |  |  |
| Thistles | Stem | Asteraceae |  | 9.49 |  |  |  |  |  | 7.41 |  |  |  |  |
| *Galium spp* | All | Rubiaceae | 43.79 | 21.17 | 25.29 | 15.38 | 22.33 | 20.00 |  | 16.05 |  |  |  |  |
| *Lobelia giberroa* | Stem | Lobeliaceae |  |  |  |  | 9.22 |  |  |  |  |  |  |  |
| *Lobelia stuhlmanii* | Bark | Lobeliaceae | 13.02 |  |  |  |  |  |  |  |  |  |  |  |
| *Lobelia stuhlmanii* | Stem | Lobeliaceae |  |  |  | 13.46 |  | 10.00 | 28.57 |  |  |  |  |  |
| *Rubus spp* | Leaf | Rubiaceae |  |  | 19.54 | 7.69 | 16.50 |  | 42.86 | 9.88 |  |  |  |  |
| *Dendrosenecio erici-rosenii* | Pith | Asteraceae | 14.20 | 16.79 |  | 19.23 |  | 20.00 |  | 4.94 |  |  |  |  |
| *Dendrosenecio erici-rosenii* | Stem | Asteraceae |  |  |  |  |  | 20.00 |  |  |  |  |  |  |
| **N feeding observations** |  |  | **169** | **137** | **87** | **52** | **206** | **10** | **14** | **81** | **0** | **0** | **0** | **0** |
| **% covered by key foods** |  |  | **88.76** | **81.02** | **86.21** | **86.54** | **81.07** | **80.00** | **85.71** | **81.48** |  |  |  |  |
| **Diet diversity** |  |  | **2.509** | **3.501** | **2.051** | **4.244** | **3.553** | **3.561** | **1.631** | **4.461** |  |  |  |  |

Supplementary Table 12. Food types-item combinations making up ~80% of the four northeast groups’ diet on days when they ranged in the ‘bamboo/mixed bamboo zone’ without consuming bamboo shoot.

| **Food item** | **Part** | **Plant family** | **SAB** | **MUH** | **KSA** | **KWI** |
| --- | --- | --- | --- | --- | --- | --- |
| ***Oldeania alpina*** | Leaf | Poaceae | 20.22 | 12.43 | 5.08 | 14.90 |
| ***Basella alba*** | Leaf | Basellaceae | 9.83 | 12.66 | 8.02 | 2.98 |
| **Thistles** | Flower | Asteraceae |  |  |  | 1.16 |
| **Thistles** | Leaf | Asteraceae | 7.58 | 11.73 | 9.63 | 3.97 |
| **Thistles** | Root | Asteraceae | 4.49 | 11.61 | 9.63 | 3.81 |
| **Thistles** | Stem | Asteraceae | 2.67 |  | 1.87 | 1.49 |
| ***Clematis simensis*** | Pith | Ranunculaceae |  |  | 9.89 | 6.29 |
| ***Discopodium penninervium*** | Pith | Solanaceae |  |  |  | 1.16 |
| ***Droguetia iners*** | Leaf | Urticaceae |  | 1.74 | 3.74 |  |
| ***Englerina species*** | Stem | Loranthaceae |  |  |  | 1.66 |
| ***Galium spp*** | All | Rubiaceae | 3.79 | 2.79 | 2.67 | 2.81 |
| ***Mimulopsis solmsii*** | Flower | Acanthaceae |  |  |  | 1.16 |
| ***Pavetta rwandensis Bridson*** | Fruit | Rubiaceae |  |  |  | 1.32 |
| ***Pleopeltis macrocarpa*** | All | Polypodiaceae |  |  |  | 5.30 |
| ***Rubus species*** | Leaf | Rubiaceae |  |  | 2.14 |  |
| ***Secamone africana*** | Leaf | Apocynaceae |  | 4.30 | 7.49 | 10.93 |
| ***Senecio maranguensis*** | Pith | Asteraceae |  |  |  | 0.99 |
| ***Solanecio manii*** | Pith | Asteraceae |  |  |  | 1.49 |
| ***Scepocarpus hypselodendron*** | Bark | Urticaceae | 25.42 | 14.05 | 13.90 | 13.91 |
| ***Scepocarpus hypselodendron*** | Leaf | Urticaceae | 6.32 | 8.71 | 7.22 | 5.46 |
| **N feeding observations** |  |  | **712** | **861** | **374** | **604** |
| **% covered by key foods** |  |  | **80.34** | **80.02** | **81.28** | **80.79** |

Supplementary Table 13. Observed diet diversity (Hill-Shannon: q=1) by vegetation zone and gorilla group sorted from right to left by group location from the furthest southwest (DUS=Dushishoze) to the furthest northeast (KWI=Kwitonda).

| **Vegetation type** | **DUS** | **PAB** | **MSK** | **KRB** | **TOB** | **SEG** | **TIT** | **NTA** | **SAB** | **MUH** | **KSA** | **KWI** |
| --- | --- | --- | --- | --- | --- | --- | --- | --- | --- | --- | --- | --- |
| **Alpine/subalpine** | 2.509 | 3.501 | 2.051 | 4.244 | 3.553 | 1.631 | 3.561 | 4.461 | - | - | - | - |
| **Meadow** | 0.480 | - | - | - | - | - | - | - | - | - | - | - |
| **Herbaceous** | - | - | - | - | - | 0.914 | - | 0.676 | 3.932 | 2.819 | 3.572 | 0.406 |
| ***Hagenia-Hypericum*** | 2.723 | 2.522 | 2.816 | 2.969 | 4.032 | 5.683 | 5.180 | 4.194 | 0.806 | - | - | - |
| **Bamboo/mixed Bamboo** | 1.177 | 1.844 | 1.479 | 2.608 | 2.897 | 4.414 | 4.494 | - | 6.662 | 8.003 | 7.779 | 8.940 |
| **Mixed forest** | - | - | 0.468 | - | - | - | - | - | - | - | 8.277 | 9.635 |
| **Out of the park** | - | 0.050 | - | 0.168 | - | 0.480 | 0.903 | - | 3.861 | 1.272 | 6.150 | 5.856 |

Supplementary Table 14. Statistical output of GLM comparing lipid proportions in key foods of the study groups (grey indicates southwest groups) with Muhoza (MUH) as reference level (Kwisanga group was excluded).

| Group compared to MUH | Est | SE | t-value | p-value |
| --- | --- | --- | --- | --- |
| DUS | -0.443 | 0.283 | -1.567 | 0.121 |
| PAB | -0.453 | 0.284 | -1.599 | 0.103 |
| MSK | -0.552 | 0.306 | -1.807 | 0.114 |
| KRB | -0.567 | 0.276 | -2.044 | **0.045** |
| TOB | -0.481 | 0.253 | -1.899 | 0.061 |
| SEG | -0.489 | 0.243 | -2.014 | **0.048** |
| TIT | -0.754 | 0.279 | -2.701 | **0.009** |
| NTA | -0.783 | 0.299 | -2.616 | **0.011** |
| SAB | -0.111 | 0.203 | -0547 | 0.586 |
| KWI | -0.190 | 0.183 | -1.040 | 0.302 |

Supplementary Table 15. Mean and range (in parentheses) percentage of nutrients (% dry matter) and metabolic energy (in kJ/g dry matter) in plant food type-items consumed by wild gorillas. CP=crude protein, L=lipids, NDF=neutral detergent fiber ADF=acid detergent fiber, TNC=total nonstructural carbohydrates

| ***Gorilla* subspecies (population)** | **Metabolic energy (kcal/g)** | **CP** | **L** | **NDF** | **ADF** | **TNC** | **Study** | **Notes** |
| --- | --- | --- | --- | --- | --- | --- | --- | --- |
| *G. gorilla gorilla* | 4.2  (2.95-5.22) | 9.2  (1.5-32.2) | 4  (0.5-20.9) | 59.9  (21.3-89.7) | 46.0  (6.0-81.1) | - | Calvert 1985 | >70% foods included |
| *G. gorilla gorilla* | - | 16.8  (0.9-25.6) | 2.6  (0.2-31.9) | - | 28.8  (4.8-61.8) | - | Rogers et al. 1990 | >70% foods included |
| *G. gorilla gorilla* | - | 11.8  (1.7-30.0) | 0.5  (0.1-1.8) | 71.7  (50.3-94.2) | 53.3  (29.0-83.8) | - | Popovich et al. 1997 | <70% foods included |
| *G. gorilla gorilla* | - | 14.3 | - | 48.9 | 34.2 | 25.3 | Lodwick & Salmi 2019 | Including major food parts (fruit, leaf, herb) |
| *G. beringei beringei* (Bwindi) | - | 9.93  (1.6-28.5) | - | 52.3  (20.6-88.9) | 31.9  (13.8-60.9) | - | Rothmann et al. 2006 |  |
| *G. beringei beringei* (Virunga) | - | 15.3  (6.6-30.0) | - | 51.3  (36.7-65.3) | - | 20.7  (10.3-34.3) | Rothmann et al. 2007 | >70% foods included |
| *G. beringei beringei* (Virunga) | 2.54  (1.84-3.45) | 13.5  (2.3-32.9) | 1.7  (0.2-16.0) | 45.7  (18.1-82.5) | 32.3  (3.8-61.0) | 28.0  (3.6-68.2) | Present study | 11.5% foods included |


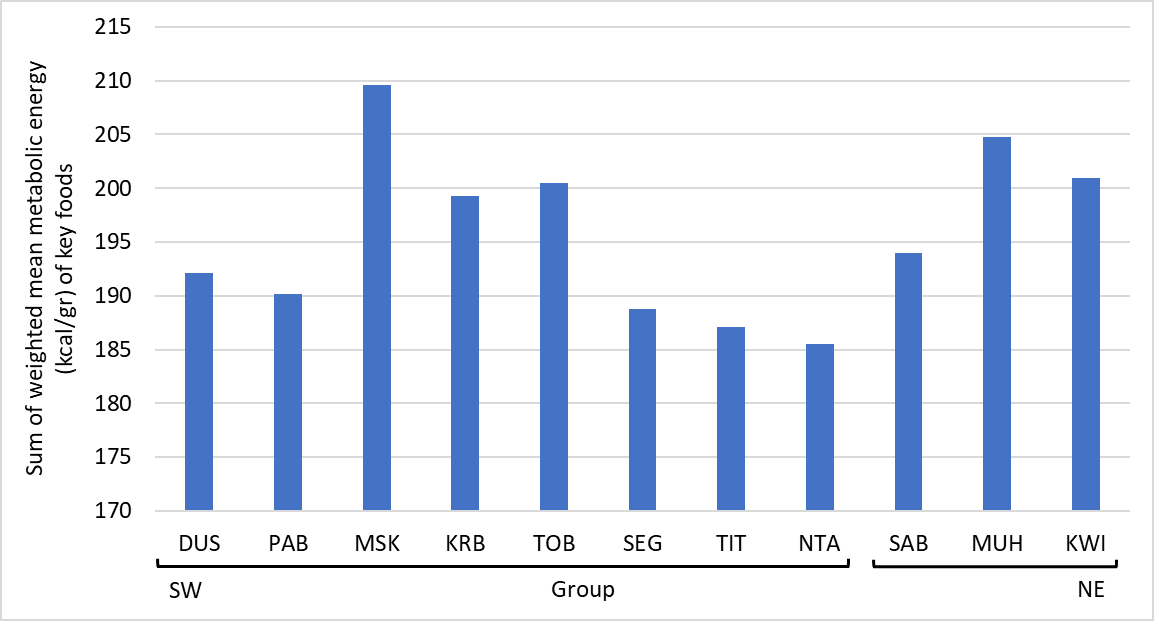


Supplementary Figure 1. Sum of weighted means of metabolic energy (in kcal/gr) of key foods (making up 80% of the diet) by each key foods’ dietary importance (% in diet) for each study group ranging in the southwest (SW) and northeast (NE) VNP. Note that foods included in the diet of KSA only make up 77.5% because *Acacia mearnsii* was not sampled for nutritional analysis.


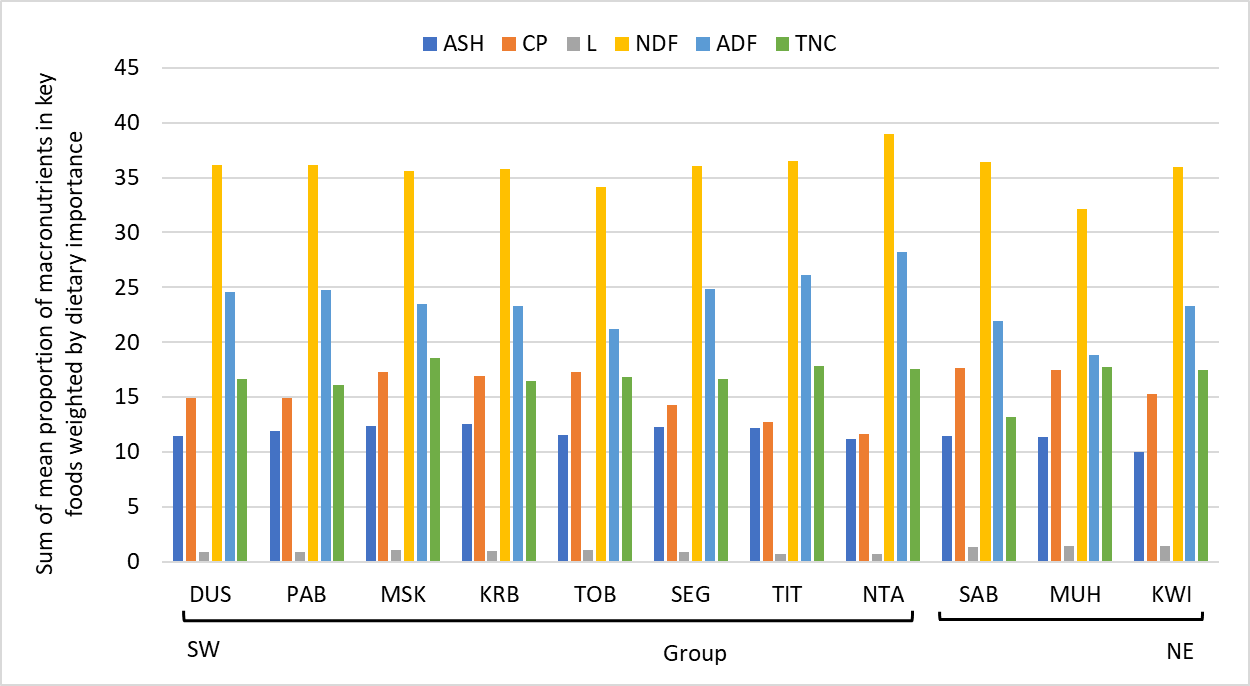


Supplementary Figure 2. Sum of mean proportion of macronutrients (ASH = total ash, CP = crude protein, NDF = neutral detergent fiber, ADF = acid detergent fiber, L= lipids, TNC = total nonstructural carbohydrates) in key foods (making up ~80% of the diet) weighted by each foods’ dietary importance (% in diet) for each study group. Note that foods included in the diet of KSA only make up 77.5% because *Acacia mearnsii* was not sampled for nutritional analysis.
